# Multiple LPA_3_ Receptor Agonist Binding Sites Evidenced Under Docking and Functional Studies

**DOI:** 10.3390/ijms26094123

**Published:** 2025-04-26

**Authors:** K. Helivier Solís, M. Teresa Romero-Ávila, Ruth Rincón-Heredia, Sergio Romero-Romero, José Correa-Basurto, J. Adolfo García-Sáinz

**Affiliations:** 1 Departamento de Biología Celular y Desarrollo, Instituto de Fisiología Celular, Universidad Nacional Autónoma de México, Ciudad Universitaria, Ap. Postal 70-600, Ciudad de México 04510, Mexico; samsonyte09@gmail.com (K.H.S.); tromero@ifc.unam.mx (M.T.R.-Á.); 2 Unidad de Imagenología, Instituto de Fisiología Celular, Universidad Nacional Autónoma de México, Ciudad Universitaria, Ap. Postal 70-600, Ciudad de México 04510, Mexico; rrincon@ifc.unam.mx; 3 Departamento de Bioquímica y Biología Estructural, Instituto de Fisiología Celular, Universidad Nacional Autónoma de México, Ciudad Universitaria. Ap. Postal 70-600, Ciudad de México 04510, Mexico; sromero@ifc.unam.mx; 4 Laboratorio de Diseño y Desarrollo de Nuevos Fármacos e Innovación Biotecnológica, Escuela Superior de Medicina, Instituto Politécnico Nacional, Plan de San Luis y Díaz Mirón S/N, Casco de Santo Tomas, Miguel Hidalgo, Ciudad de México 11340, Mexico; corrjose@gmail.com

**Keywords:** lysophosphatidic acid, LPA, LPA_3_ receptor, oleoyl-methoxy glycerophosphothionate, OMPT, biased agonism, ligand docking

## Abstract

Comparative studies using lysophosphatidic acid (LPA) and the synthetic agonist, oleoyl-methoxy glycerophosphothionate (OMPT), in cells expressing the LPA_3_ receptor revealed differences in the action of these agents. The possibility that more than one recognition cavity might exist for these ligands in the LPA_3_ receptor was considered. We performed agonist docking studies exploring the whole protein to obtain tridimensional details of the ligand–receptor interaction. Functional in cellulo experiments using mutants were also executed. Our work includes blind docking using the unrefined and refined proteins subjected to hot spot predictions. Distinct ligand protonation (charge −1 and −2) states were evaluated. One LPA recognition cavity is located near the lower surface of the receptor close to the cytoplasm (Lower Cavity). OMPT displayed an affinity for an additional identification cavity detected in the transmembrane and extracellular regions (Upper Cavity). Docking targeted to Trp102 favored binding of both ligands in the transmembrane domain near the extracellular areas (Upper Cavity), but the associating amino acids were not identical due to close sub-cavities. A receptor model was generated using AlphaFold3, which properly identified the transmembrane regions of the sequence and co-modeled the lipid environment accordingly. These two models independently generated (with and without the membrane) and adopted essentially the same conformation, validating the data obtained. A DeepSite analysis of the model predicted two main binding pockets, providing additional confidence in the predicted ligand-binding regions and support for the relevance of the docking-based interaction models. In addition, mutagenesis was performed of the amino acids of the two detected cavities. In the in cellulo studies, LPA action was much less affected by the distinct mutations than that of OMPT (which was almost abolished). Therefore, docking and functional data indicate the presence of distinct agonist binding cavities in the LPA_3_ receptor.

## 1. Introduction

Lysophosphatidic acid (LPA) is a bioactive lipid that exerts many of its actions through six G protein-coupled receptors (GPCRs) that constitute the LPA subfamily [1,2,3]. Among them is the LPA_3_ receptor, which mainly couples to G_αi/o_ and G_αq/11_, participating in diverse signaling events that lead to various physiological and pathophysiological effects [4,5]. Structurally, the LPA_3_ receptor is a membrane protein of 353 amino acids containing seven transmembrane domains (TM), which are connected by three intracellular (IL) and three extracellular (EL) loops; it comprises an extracellular amino terminus and an intracellular carboxyl end [2,4,5]. Though it has not been crystallized yet, it has a similarity of approximately 53% with the LPA_1_ receptor, the best-studied member of this group [3,6,7,8]. It is worth mentioning that the domains that regulate LPA_1_ receptor function are not conserved in the LPA_3_ receptor; these include a dileucine motif, a mono ubiquitination residue, and a PDZ pattern, among others [5,9]. A structural analysis of the LPA_3_ receptor showed that it conserves motifs/domains typical of the A family of GPCRs, which comprise CWxP, EHR, PIF, and NPxxY (names correspond to the one-letter code of the constituting amino acids; see [10]), each having a specific function during and after agonist attachment [2,5,10,11].

It is generally accepted that the receptor’s recognition cavities are flexible and that ligand binding promotes conformational changes that impact their spatial configuration; biochemical and biophysical techniques to address ligand–receptor interactions in their structural and dynamic aspects were critically reviewed by Kaiser and Coin [12]. These dynamic events allow displacements of the intracellular loops and the TM regions, which promote G protein recruitment and the exposure of sites that can be phosphorylated by distinct protein kinases, including G protein-coupled receptor kinases (GRKs) and second messenger-activated protein kinases [11,12,13,14,15]. Receptor phosphorylation favors β-arrestin–receptor interaction, triggering its internalization and subsequent signaling events [16,17,18]. Determining how a ligand interrelates with a given receptor provides fundamental insights into the search for drugs that could effectively regulate its activity and the participating mechanisms. In this sense, molecular docking simulations have become a method that allows for better knowledge of the receptor regions in which the ligand could bind, suggesting the possible residues involved [19].

Pioneer structural work with the LPA_3_ receptor identified amino acids that interact with the polar head of LPA [7,20,21,22,23]. When these residues were mutated, their function was altered, particularly those in the TM domains 3, 4, 5, and 7, mainly involving the following sites: Arg105 (3.28), Gln106 (3.29), Trp153 (4.64), Arg209 (5.38), and Lys296 (7.35) (in parentheses are the Ballesteros and Weinstein [24] numbering scheme, which is based on the presence of highly conserved residues in each of the seven transmembrane domains; it consists of two numbers, the first denoting the helix and the second the residue position relative to the most conserved residue. For the LPA_3_ receptor, these numbers can be obtained from the server: https://gpcrdb.org/protein/lpar3_human/ [25] accessed 12 March 2024). It is relevant that the mutation of residues Trp153 (4.64) and Lys296 (7.35) directly impacts ligand binding and the activation of the LPA_3_ receptor, i.e., LPA potency and efficacy were decreased in RH7777 cells transfected with mutant receptors when compared to those expressing the wild-type [22].

No specific agonist for the LPA_3_ receptor subtype is available since most also activate the LPA_1_ and LPA_2_ receptors. A ligand that shows high potency for LPA_3_ is 1-oleoyl-2-methyl-sn-glycero-3-phosphothionate (OMPT) [26]. OMPT and related analogs exhibit affinity for different LPA receptors, but they have a weak selectivity for the LPA receptor subtypes [27,28,29]; despite these selectivity problems, OMPT remains a reference for studies on LPA_3_ receptors. To our knowledge, the binding site for OMPT in LPA receptors has not been explored.

It was recently reported that LPA and OMPT exhibit marked pharmacodynamic differences in LPA_3_-expressing cells [30]. In particular, two findings were astonishing: firstly, OMPT exerts its effects within two very distinct concentration ranges (1–10 nm and >100 nM), and secondly, LPA induced a “receptor refractory state” that blocks calcium response to re-stimulation with this natural agonist; however, under the same conditions, OMPT was able to produce a conspicuous response [30]. Both groups of data suggest the possibility that distinct binding cavities might exist for these agonists in the LPA_3_ receptor: one showing a very high affinity for OMPT and another one, likely shared by LPA and OMPT (which are structural analogs), with a lower affinity for both agonists in the range of 100–300 nM [30]. Intrigued by such findings and lacking structural information on the OMPT-LPA_3_ receptor interaction, we performed ligand–receptor docking studies to find insights into their protein binding pocket(s). The docking examination and the hot spot predictions indicate the presence of at least two distinct ligand recognition cavities, which might explain the observed functional responses to OMPT. In addition, receptor mutants were constructed and expressed, and their response to these agonists was studied. One of the mutants showed activity in reaction to LPA and essentially no effect on OMPT, which is consistent with the proposal of multiple binding pockets for these ligands [30]. This report documents distinct agonist identification cavities via docking simulations and in cellulo experiments with receptor mutants. The findings open new experimental possibilities and might help better understand LPA_3_ receptor–ligand interaction and aid in developing new agonists and antagonists of potential therapeutic use.

## 2. Results

### 2.1. LPA_3_ Receptor Model Without Refinement

The LPA_3_ receptor has not been crystallized yet; therefore, its most likely 3D structure was obtained using different servers for configuration prediction (additional information is provided in Section 4.1). Supplementary Table S1 enlists the values obtained using these servers and programs. Protein arrays from the distinct patterns were subjected to sequence alignments (Supplementary Figure S1), and the Swiss Model was preferred because the best structural score values were obtained, and the resulting sequence was identical to the UniProt Q9UBY5 entry.

The unrefined 3D pattern from the Swiss Model program was employed to determine the binding parameters (free energy values (ΔG) and residue interaction) for the LPA_3_ receptor agonists’, LPA and OMPT (a 126 Å^3^ grid box centered on the protein with (x, y, and z), coordinates in the grid center for both agonists: 6.014, −0.124, and −2.385). Molecular docking simulation showed that the LPA_3_ receptor recognition cavities for LPA and OMPT were different (Supplementary Table S2), and the binding energy value was lower for OMPT than for LPA. The residues participating in ligand–receptor interaction are also indicated in Supplementary Table S2; please notice that the amino acids involved are listed in the order indicated by the program, considering their importance in the ligand–receptor interaction. Supplementary Figure S2 depicts such interrelations using the unrefined model. OMPT (red) associates with amino acids located in TM regions 2 and 7 and the EL 1, 2 (we name this broad region Upper Cavity; see Supplementary Figure S2), whereas LPA (blue) reached a distinct site (broad region named Lower Cavity) of the LPA_3_ receptor structure with molecules preferentially located in IL 2 and IL 3, very close to the cytoplasm (Supplementary Figure S2). The cartoon shown in Supplementary Figure S3 depicts the LPA-interacting residues (blue) and OMPT-associating amino acids (red) (notice the remarkably different distribution of such binding cavities).

The 2D maps of the ligand–receptor interrelations obtained from the Discovery Studio program are also shown (Supplementary Figures S4 (LPA) and S5 (OMPT)). This methodology allows for adaptations of the ligand to obtain the best possible interaction with the receptor and performs the necessary adjustments to achieve the most favorable association; it also permits determining the agonist orientation, identifying the receptor’s amino acids involved, and defining the type of interaction (Supplementary Table S2). Notice that the phosphate group of LPA is fully protonated (i.e., net charge 0; Supplementary Figure S4), but in contrast, the phosphorothioate group of OMPT is partially deprotonated (net charge −1; Supplementary Figure S5) when interacting with the distinct LPA_3_ receptor–ligand recognition cavities.

The polar head groups of agonists such as LPA and OMPT seem important when these ligands bind to LPA_3_ receptors and for their activation [7,20,21,22,27]. LPA’s phosphate and OMPT’s phosphorothionate can be protonated (net charge 0), partially deprotonated (net charge −1), or fully deprotonated (net charge −2) at distinct pH values. Experimentally defined pKa values for these compounds have not been reported. Only those of the analog, phosphatidic acid, are available (pKa1 = 3.0 and pKa2 = 8.0; https://avantiresearch.com/tech-support/physical-properties/ionization-constants; accessed 28 March 2025). Therefore, the pKa values for the ligands were estimated using the software available from Chemaxon (https://chemaxon.com/calculators-and-predictors; accessed 28 March 2025). These pKa values were obtained with a quantitative structure–activity relationship (QSAR) model, which considers the ionizable states’ molecular environment and calculates their corresponding dissociation constants. Considering the pKa values for OMPT (pKa1 = 1.97 and pKa2 = 6.41), at a pH of 7.4, the distinct protonation species present are charge 0, <0.1%; charge −1, 9.28%; and charge −2, 90%. For LPA (pKa1 = 1.25 and pKa2 = 6.27) at pH 7.4, the distinct species present are charge 0, <0.01%; charge −1, 6.9%; and charge −2, 93%. The docking simulations using the unrefined receptor structure employed the protonated agonist species (charge 0). For all the following simulations, the three molecular species were employed (i.e., species with net charges 0, −1, and −2).

### 2.2. LPA_3_ Refined Model

**Figure 1:**
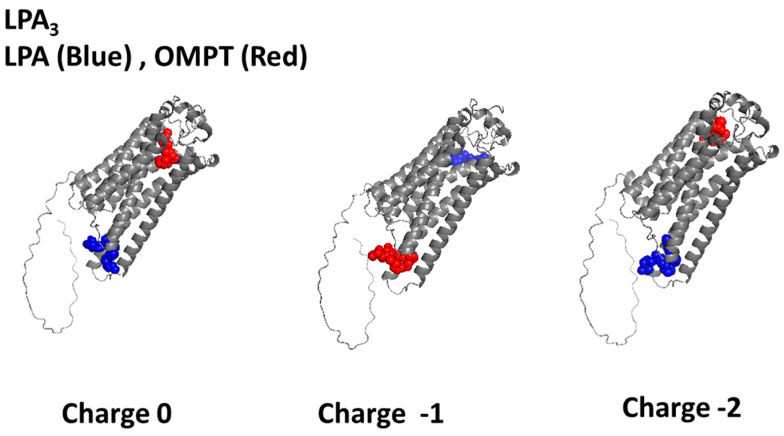
Three-dimensional images resulting from the LPA_3_-LPA (blue) and LPA_3_-OMPT (red) docking analysis using PyMol, version 3.0. The refined structure was employed. Ligand net charges are indicated.

The molecular docking studies of protonated (charge 0) LPA (Figure 1; blue) show that it occupied the Lower Cavity of the receptor, interacting with the IL 2 and 3, which, like in the LPA_3_ unrefined model, was very close to the cytoplasm. On the other hand, OMPT (charge 0) (Figure 1; red) associates with the amino acids located in the Upper Cavity, including TM 5, 6, 7, and 8 as well as extracellular loop 3 (Figure 1). Interestingly, simulation with both charge −1 ligands resulted in the inversion of the agonist position in these cavities (Figure 1). Simulation with charge −2 derived in interaction patterns similar to those observed with the uncharged ligands (Figure 1). Table 1 (for LPA) and Table 2 (for OMPT) provide the ΔG values and the list of interacting amino acids defined for the distinct conditions. Simulation with LPA (charge −2) resulted in a more negative ΔG value compared to the other conditions (Table 1); in contrast, modification of OMPT charge had only a minor impact on these parameters (Table 2). Cartoons showing the interrelating amino acids under these conditions are presented in Supplementary Figure S6, and the 2D schemes showing the interacting residues and the type of association are presented in Supplementary Figure S7.

### 2.3. Trp102-Focused Model

**Figure 2:**
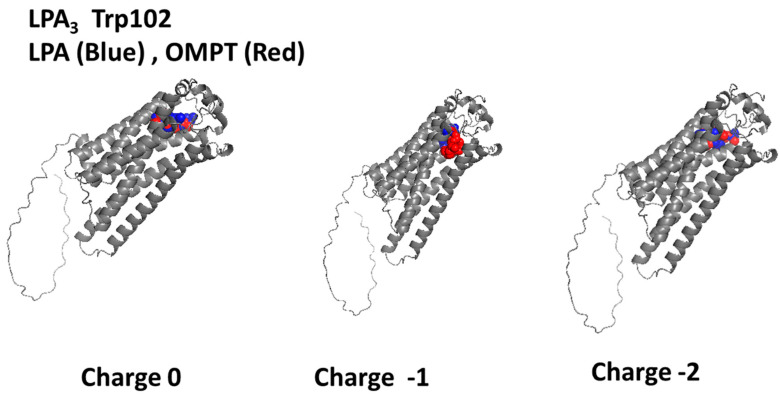
Images resulting from the docking analysis of the refined 3D structure of LPA_3_ receptor (Trp102 focused) interacting with LPA (blue) and OMPT (red). Ligand net charges are indicated.

Trp102 is located near the Upper Cavity or the LPA_3_ receptor configuration (Supplementary Figure S8). As previously mentioned, according to a 3D structural analysis with PyMol, this residue was consistently found in the hot spot predictions and visualized as exposed and centered in a cavity. Trp102-focused docking was performed for LPA and OMPT with distinct ligand protonations as indicated. Independently of the molecule charge, both agonists interacted with the receptor’s Upper Cavity (Figure 2). The uncharged ligands interrelated with the TM regions 1, 2, and 7. LPA (charge −1) interconnect with TM domains 1, 2, 3, and 7 and EL2, whereas OMPT (charge −1) binds to amino acids in TM domains 3, 5, 6, and 7 and EL 3. Simulations with both charge −2 ligands lead to their interaction with residues in TM 1 and 7 and EL 2. Cartoons showing the participating amino acids under these conditions are presented in Supplementary Figure S9, and the 2D schemes showing the interacting residues and their type of association are illustrated in Supplementary Figure S10. For LPA (Charge −2), Trp102-focused docking resulted in more negative ∆G values (Table 1); similarly, OMPT (charges −1 and −2) also derived more favorable thermodynamic values (Table 2). The cartoons and the 2D images (Figures S9 and S10) evidenced that both ligands shared some amino acids under these conditions.

### 2.4. Tyr293-Focused Model

**Figure 3:**
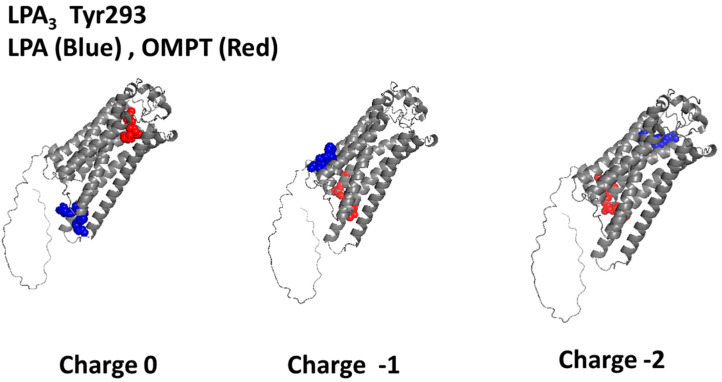
Images resulting from the docking analysis of the refined 3D structure of the LPA_3_ receptor (Tyr293 focused) interacting with LPA (blue) and OMPT (red). Ligand net charges are indicated.

The localization of Tyr293 in the LPA_3_ structure is shown in Supplementary Figure S8. This residue participates in the attachment of the LPA antagonist, Kil6425, and an extensive series of pharmacological agents used in therapeutics [31]. Figure 3 presents the non-bond interaction of LPA (blue) and OMPT (red) with the LPA_3_ receptor when the docking analysis is focused on Tyr293. Using ligands with charge 0 for the docking simulation showed that the LPA binding cavities were located near the cytoplasm in the intracellular loops 1, 2, and 3. OMPT–LPA_3_ receptor interaction occurred in the Upper Cavity involving TM domains 1 and 7 and EL 2 and 3. When simulations were performed using both ligands with charge −1, the agonists interrelated with the lower part of the receptor (LPA) IL1, IL2, and TM 4; for OMPT binding residues at IL3, Helix 8 and TM 7 played a role. With the receptor focused on Tyr 293, both molecules with charge −2 interacted with a pattern opposite to that observed at charge 0. Cartoons illustrating the partaking amino acids under these conditions are presented in Supplementary Figure S11, and the 2D schemes showing the participating residues and their type of association are depicted in Supplementary Figure S12. Table 1 shows that LPA (charge −2) exhibited a relevant negative ∆G value. OMPT charge causes minor modifications of these thermodynamic parameters.

### 2.5. Membrane-Inserted LPA_3_ Receptor Structural Model

**Figure 4:**
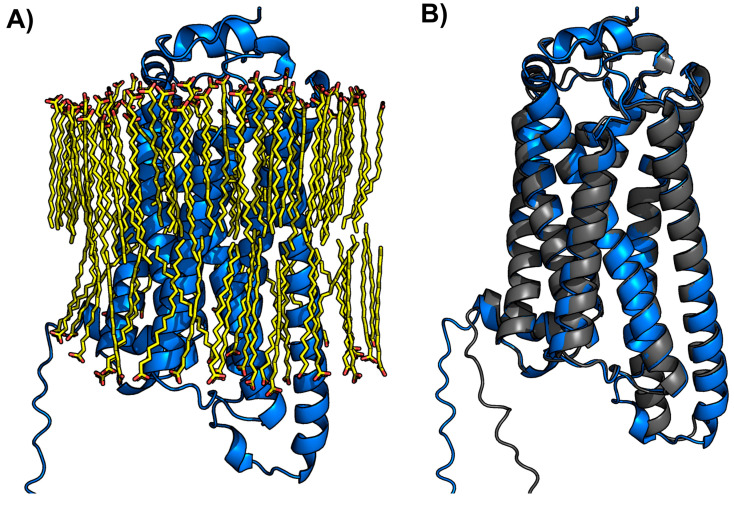
(**A**) LPA_3_ model in a membrane-mimicking environment (composed of ~100 lipid molecules shown in yellow). (**B**), structural comparison of LPA_3_ models performed in the presence of bilayer-like structure (blue) and without it (gray) (Cα RMSD = 0.673 Å over 281 residues).

Since the LPA_3_ receptor is a membrane protein, it was modeled in the presence of lipid molecules to mimic a membrane environment. The quality of the model was adequate, with a predicted TM-score (pTM) of 0.82 and an interface-predicted TM-score (ipTM) of 0.79, supporting the reliability of the structure and its correct embedding within the membrane. Remarkably, the resulting model showed a bilayer-like arrangement of lipids with the receptor correctly inserted. The AlphaFold3-generated model included the automatic organization of approximately 100 lipid molecules, forming a bilayer-like structure with the receptor correctly inserted into the membrane (Figure 4A). These data indicated that AlphaFold3 identified the transmembrane regions of the sequence and co-modeled the lipid environment accordingly.

Additionally, comparing the models generated with and without the membrane context yielded a Cα RMSD of 0.673 Å over 281 residues, indicating that both models adopt almost the same conformation (Figure 4B). The structural similarity between the membrane-embedded and non-membrane models validates the data obtained without considering the membrane. This approach markedly reduces computational time and costs and should be considered after validation, as in this case, in studies with other receptors.

**Figure 5:**
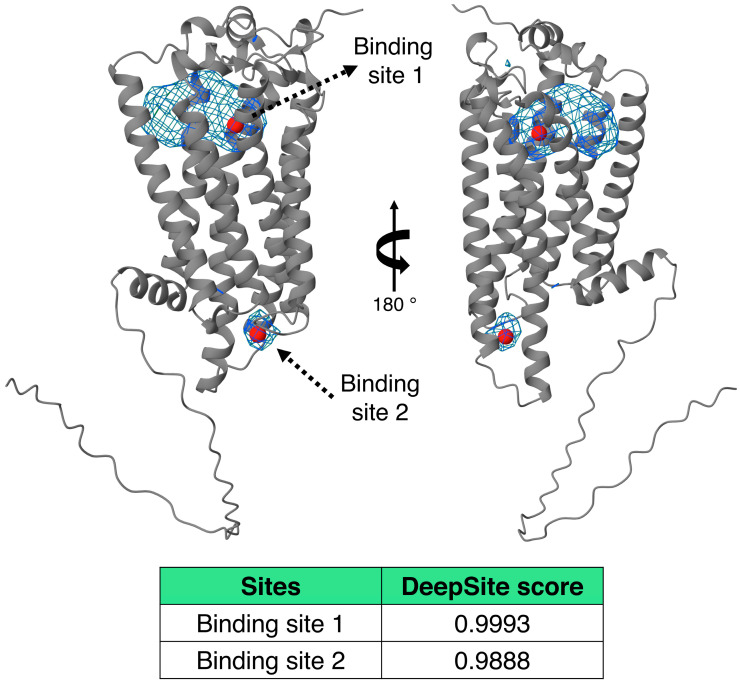
Predicted binding sites on the LPA_3_ receptor structure. The two main binding pockets identified by DeepSite are shown as blue mesh surfaces. Red spheres indicate the geometric centers of the predicted binding sites. Two views of the receptor (rotated 180°) are shown to provide a complete perspective of the binding site locations. The table summarizes the binding site scores calculated by DeepSite.

To further support the identification of ligand-binding sites, we applied DeepSite to the LPA_3_ receptor model. This analysis predicted two main binding pockets overlapping with the top-ranked docking poses obtained in our study. These results provide additional confidence in the predicted ligand-binding regions and support the relevance of the docking-based interaction models. The predicted pockets and their scores are shown in Figure 5.

### 2.6. In Cellulo Signaling Experiments

**Figure 6:**
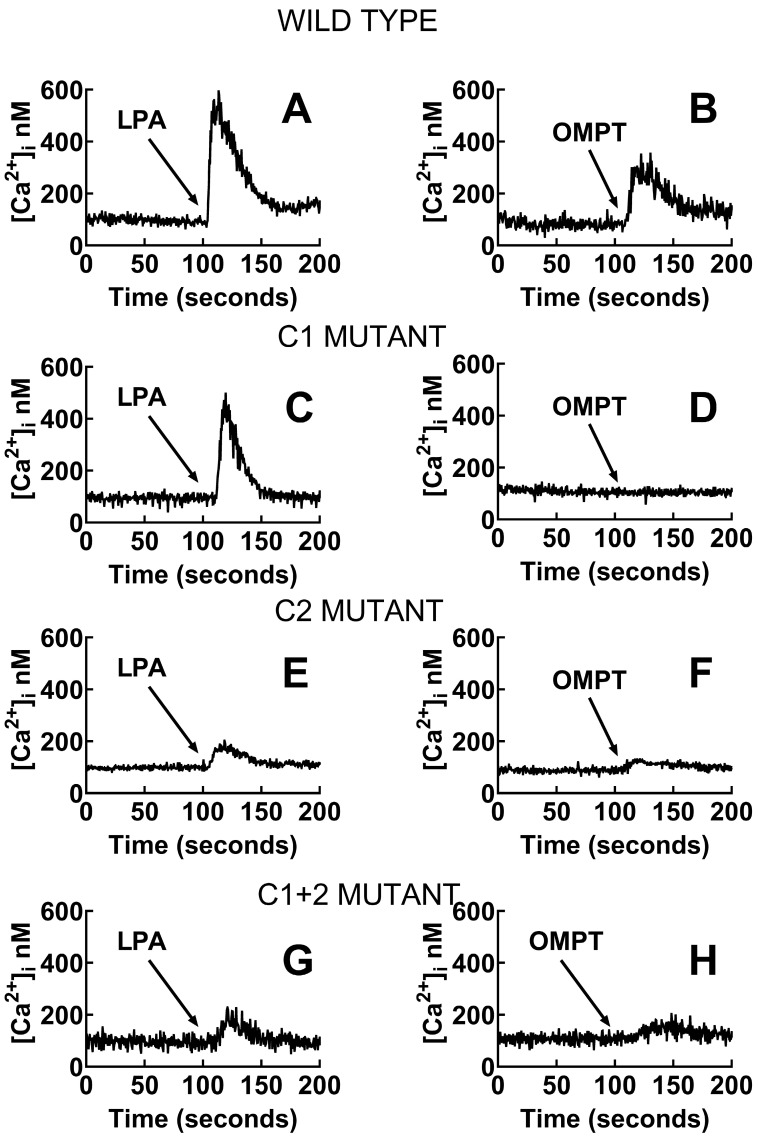
Representative calcium tracings were obtained in cells expressing the wild-type LPA_3_ (**A**,**B**) receptors or mutants with substitutions in Cavity 1 (C1 mutant; **C**,**D**), Cavity 2 (C2 mutant; **E**,**F**), or both cavities (C1 + 2 mutant; **G**,**H**). Cells were stimulated (arrow) with 1 µM LPA (**right**) or 1 µM OMPT (**left**).

**Figure 7:**
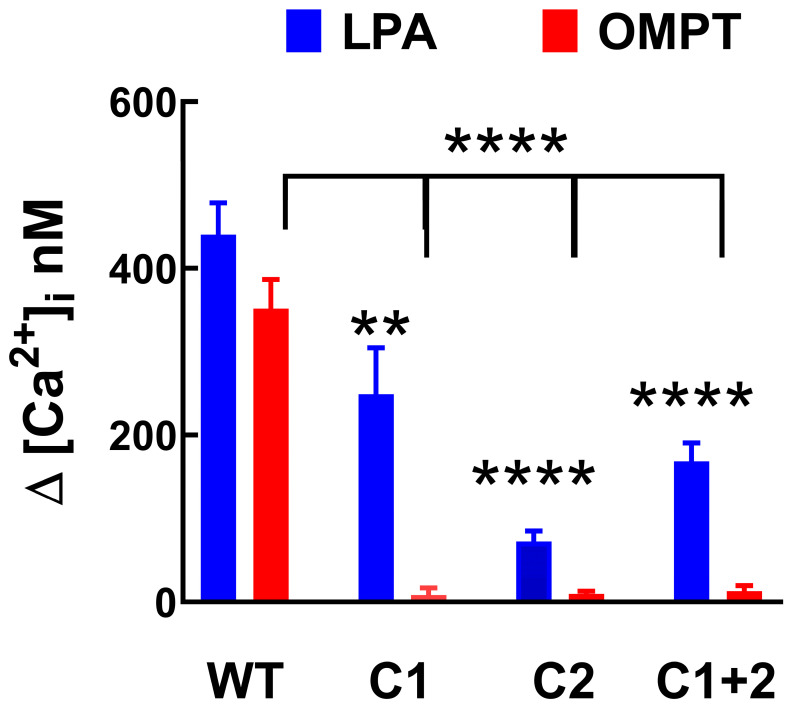
Calcium tracings were obtained in cells expressing the wild-type LPA_3_ receptors or mutants with substitutions in Cavity 1 (C1 mutant), Cavity 2 (C2 mutant), or both Cavities (C1 + 2 mutant). Cells were stimulated with 1 µM LPA or 1 µM OMPT. The means are plotted, and vertical lines indicate the SEM of 4-5 experiments performed on different days using distinct cell cultures. ** *p* < 0.01, and **** *p* < 0.0001 vs. cell expressing the wild-type receptor,.

**Figure 8:**
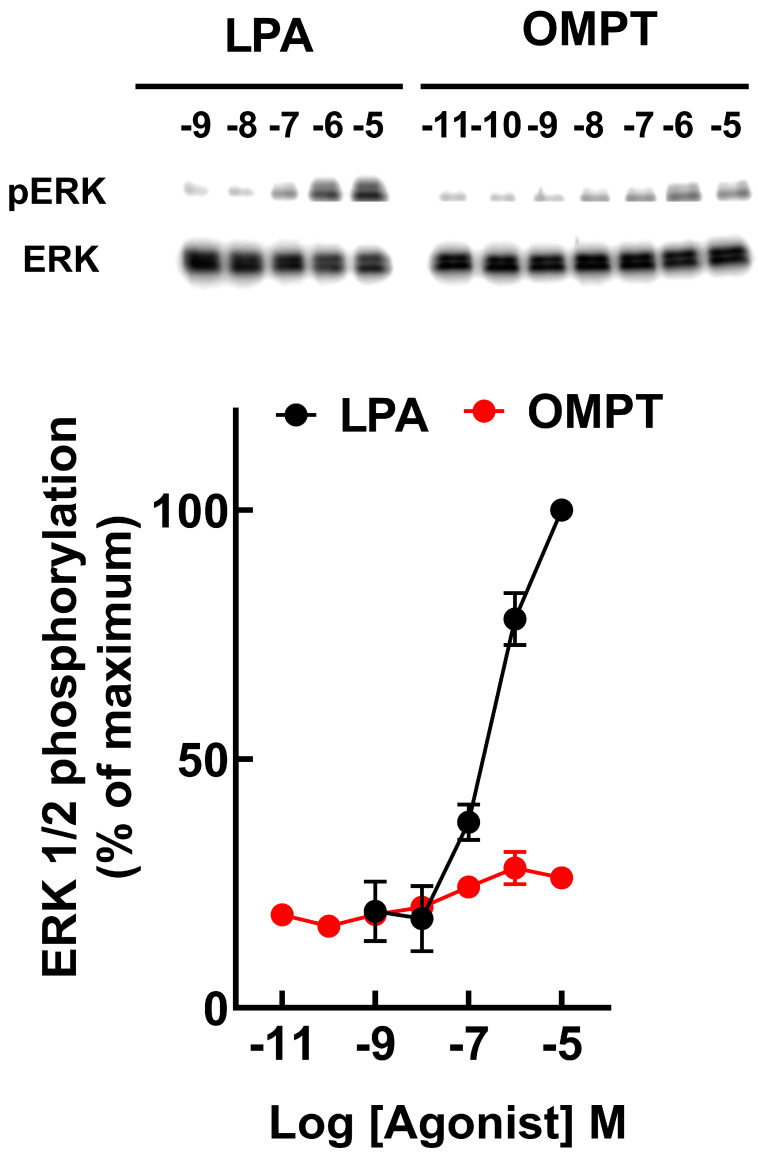
The effect of LPA and OMPT on ERK 1/2 phosphorylation in cells expressing the LPA_3_ receptor mutants with substitutions in Cavity 1. Cells were stimulated with 1 µM LPA or 1 µM OMPT. The means are plotted, and vertical lines indicate the SEM of 4–5 experiments performed on different days using distinct cell cultures.

We have previously shown that in transfected but not induced cells, LPA triggered a minimal, barely detectable response, but when the LPA_3_ receptors were induced, the response was much bigger [32]. We confirmed this observation and extended it to OMPT (Supplementary Figure S13); these effects were blocked by the LPA_1,3_ antagonist, Ki16425 [33]. The data evidenced that the observed actions were mediated through the expressed LPA_3_ receptor. Mutant receptors in which the ligand-interacting residues (unrefined model) in the cavities were substituted for alanine were expressed, and their calcium response to 1 µM LPA and 1µM OMPT was determined. The reaction of cells manifesting the wild-type receptor was considered the reference and was compared to the mutants in the Upper Cavity, Lower Cavity, and both. Representative calcium tracings were presented in Figure 6, and the data analysis is shown in Figure 7. In the control cells, as anticipated, 1 µM LPA or 1 µM OMPT triggered an almost immediate increase in intracellular calcium. In contrast, a very weak response to OMPT was observed with the distinct mutants. The reaction to LPA was diminished but not blocked, and the Upper Cavity mutant showed a robust effect. These data indicate that some residues are much more relevant for the action of OMPT than for that of LPA. Similar findings were obtained when ERK 1/2 phosphorylation was studied. OMPT is more potent than and similarly effective to LPA to induce ERK 1/2 phosphorylation [26,30]. Using cells expressing the Upper Cavity mutant, it was observed that the action of OMPT was essentially abolished, whereas a clear concentration-dependent response to LPA was detected (Figure 8).

## 3. Discussion

The present work was designed to define if LPA and OMPT bind to a single cavity or different agonist recognition pockets in the LPA_3_ receptor. The possible existence of more than one ligand-identification region was suggested in experiments using cultured cells [30]. Blind docking studies were performed using the predicted configuration of LPA_3_ (UniProt, Q9UBY5 entry). Three-dimensional structures of refined and focused (to Tyr293 and Trp102) docking analyses were carried out employing the distinct protonation states of the ligands. As indicated, Tyr293 was identified as a critical amino acid for several agents interacting with the LPA_3_ receptor [31], and Trp102 was consistently detected in the hot spot prediction.

In our work, we observed the docking of LPA and OMPT to two principal LPA_3_ receptor regions named, for this work, Upper and Lower Cavities. This pattern was noticed in both the unrefined and refined docking analysis and with changes in the net charge of the ligands. In fact, under most of these conditions, LPA was localized in the Lower Cavity. This location was reversed when a charge of −1 was assigned to both ligands. Previous work [7,20,21,22,23] has detected a central binding cavity with emplacement similar to the Upper Cavity reported here. In these studies, a box of 60 Å^3^ was employed, whereas in our work, we used a 126 Å^3^ box and a grid space of 0.375 Å^3^. Therefore, it seems likely that space limitations (i.e., using a box of 60 Å^3^) might have impeded the Lower Cavity detection. Avoiding expanse restrictions has positive and negative aspects. When the ligand recognition cavity(s) is not known, it seems convenient to explore the entire protein surface by docking, a procedure named “blind docking”, which implies using a large box [34]; however, focusing on predicted binding regions is known to improve docking accuracy and efficiency [34].

It should be mentioned that the Upper Cavity is in the TM core of the receptor, where most agonists associate with class A GPCRs. The Lower Cavity observed in this work included ligand interaction with ICLs and hardly any contact with the TM domains, which is an uncommon feature. This Lower Cavity might represent a gating point for the molecules rather than an agonist-activating region. However, with the current information, determining the functional relevance of these cavities is impossible. The operational data obtained with the mutants of these pockets indicate that amino acid substitution in any of them markedly altered the action of OMPT and, to a much lesser extent, that of LPA. These findings support the idea that the effect of these two agonists does not involve the same receptor associations but rather that distinct residues participate in receptor activation.

Previous work, also using docking/computational modeling and mutants, identified that the LPA_3_ receptor interacted mainly with the polar head of LPA at Arg105 (3.28), Gln106 (3.29), Trp153 (4.50), Arg209 (5.60), Arg276 (7.36), and Lys275 (7.35) [7,20,21,22,23]. These amino acids were considered critical for LPA binding to the receptor, and their mutation decreased agonist efficacy and potency; as expected, such modifications eliminated ionic interactions in the modeled LPA_3_-ligand prototype [7,20,21,22,23]. It is important to mention that the same amino acids were identified in our docking studies. The residues detected were Arg276 (9 times), Arg105 (4 times), Lys275 (3 times), Gln106 (2 times), and Trp153 (1 time); this information includes data for LPA and OMPT. Other interacting amino acids frequently found in the docking simulations for both ligands include Val33 (9 times), Trp25 (7 times), Trp277 (7 times), Leu280 (7 times), Leu86 (7 times), and Leu283 (6 times), among others.

The similarities of our present findings with those published earlier [7,20,21,22,23] suggest the possibility that the LPA binding site observed in the Upper Cavity might correspond to that previously determined [7,20,21,22,23]. No prior docking data for LPA_3_–OMPT interaction has been published. Our findings indicate that OMPT occupies the same Upper Cavity as LPA. Interestingly, the functional information formerly published [28] and that using mutants presented here strongly suggest that differences among these agonist-recognizing regions exist; therefore, they should be considered distinct, although some interacting residues could be shared. Exploration of the LPA_3_ receptor cavities, using PyMol, showed many capable of ligand binding (Supplementary Figure S14). The localization of the agonists was included in the analysis, employing the Trp102 docking simulations (charges −1 and −2). It can be observed that LPA and OMPT occupy close but not identical positions; in addition, it seems likely that molecules with distinct charges (i.e., mixtures of agonists with charges −1 and −2) might interact with LPA_3_. Very recent data using single-particle cryo-EM structures of human S1P_1_ receptor bound to Siponimod (a synthetic agonist) and heterotrimeric Gi complexes as well as human LPA_1_ receptor and Gi complexes in the presence of LPA indicated that the ligands adopt different conformations to interact with their cognate GPCRs [35]. Such distinct molecule configurations might participate in the agonist-induced LPA_3_ activation. Similar structural approaches likely would be required to define the precise residues that interact with the different ligands and their diverse conformations. In the present work, data showed that the LPA_3_ receptor has two primary ligand binding sites, but it should be considered that the receptor might have other binding sites, even within the same cavities, that could favor distinct receptor conformations impacting their function. The present findings open new research possibilities to explore this crucial step in LPA_3_ receptor signaling, considering the physiological and physiopathological events in which it is involved [4,5].

## 4. Materials and Methods

### 4.1. Modeling of LPA_3_ Receptor Structure, Gene ID:23566

The LPA_3_ receptor has not yet been crystallized. Therefore, we were forced to predict its most likely 3D structure using the amino acid sequence obtained from the Uniprot database (Q9UBY5 entry) (https://www.uniprot.org/uniprotkb/Q9UBY5/entry; accessed 5 August 2024). Three of the most frequently utilized servers for structure prediction were employed: SWISS-MODEL (https://swissmodel.expasy.org/: accessed 5 August 2024), I-TASSER (https://zhanggroup.org/I-TASSER/, version 5.2; accessed 3 August 2024), and AlphaFold2 database (https://alphafold.ebi.ac.uk/ accessed 5 August 2024). Supplementary Table S1 enlists the values obtained using these servers and programs. Subsequently, protein arrays from the three models were subjected to sequence alignments (Supplementary Figure S1) employing the PROMALS3D server (http://prodata.swmed.edu/promals3d/promals3d.php; accessed 6 August 2024) to determine the gaps or residue insertions that improve the arrangement identity with the 3D protein templates during the folding of the molecule. Although the data obtained showed that the 3D construction built with AlphaFold2 was the best evaluated structurally, the alignment revealed that several mutations or deletions improved the 3D configuration quality, possibly eliminating residues that can interact with the ligands. Therefore, the Swiss Model was preferred because the best values were estimated by ERRAT and a second evaluation of Ramachandran maps; additionally, the sequence obtained was identical to the UniProt Q9UBY5 entry.

### 4.2. Refinement and Validation of the LPA_3_ Receptor 3D Structure

A protein 3D structure refinement was carried out to perform accurate docking simulations. The arrangement obtained from the Swiss Model server was subjected to molecular refinement using ReFOLD (https://www.reading.ac.uk/bioinf/ReFOLD/ReFOLD3_form.html; version 3.0; accessed 10 August 2024). The refined protein with the best score was used for the docking study to explore receptor cavities capable of ligand recognition.

### 4.3. Search for Ligand-Binding Cavities on the LPA_3_ Receptor

The hot spot prediction was performed to identify possible ligand-recognition cavities using the BetaCavityWeb (http://voronoi.hanyang.ac.kr/betacavityweb/; accessed 28 March 2025) and the Fpocketweb (http://durrantlab.com/fpocketweb; accessed 28 March 2025) servers. The protein from the Swiss Model server as well as that from ReFOLD were used. Both servers calculate these cavities for a given molecular structure and a defined spherical probe and report their geometrical properties, such as volume, boundary area, buried area, and other parameters described on their websites. In proteins, cavities can be predicted mainly at the barrel end built by an alpha secondary structure, a pattern observed in the refined and unrefined models.

### 4.4. LPA and OMPT Docking at the LPA_3_ Receptor Binding Sites

The 3D configurations of the ligands studied were obtained from PubChem (ID 5,497,152 for LPA and ID 16,078,994 for OMPT), and their molecular structure minimization was carried out using the Avogadro program (https://avogadro.cc/docs/tools/auto-optimize-tool/; version 1.XX; accessed 6 August 2024), which allows the net inter-atomic force on each atom to be acceptably close to zero, and the position on the potential energy surface is such that the improved structures correspond to the configurations found in nature. The lengths and angles of the links and junctions and their geometry were appropriated and transmitted with the optimized energy.

After ligand structure minimization, a blind docking study of the native protein (unrefined) was performed, allowing us to explore the whole molecule. It is worth noticing that it has been reported that Tyr293 participates in the binding of the LPA antagonist, Kil6425, as well as of an extensive series of pharmacological agents used in therapeutics [22]. Residue Trp102 was also particularly interesting because it was consistently found in the hot spot predictions and visualized as exposed and centered in a cavity according to 3D structural analysis with PyMol, Molecular Graphics System, Version 3.0 Schrödinger (https://www.pymol.org/; accesses 14 September 2024). Therefore, focused docking simulations on the alpha carbon of Tyr293 and Trp102 were also performed. Subsequently, the files of ligands and protein employed to perform the docking analysis were obtained using the program Autodock tools 1.5.6 Sep_17_14 (https://autodocksuite.scripps.edu/adt/; accessed 3 August 2024). Autogrid4 and Autodock4 programs procured the grid maps and docking results. The PyMol program was also employed to explore the possible ligand-binding cavities in the LPA_3_ receptor.

Blind docking simulations consist of adjusting the grid box (search area) to the maximum expanse the program allows, i.e., 126 Å^3^ and a grid space of 0.375 Å^3^, centered by default on the proteins. All simulations were performed using the LINUX operating system (Centus) and the Lamarckian genetic algorithm, which was chosen to search for the best ligand configurations coupled to the LPA_3_ receptor. This process generates the most probable results of the ligand conformations within the receptor search area, with a maximum number of 1 × 10^7^ energy evaluations and a population of 100 randomized individuals (represented by the 100 best evaluations). For the focused docking, the grid box was 60 Å^3^ with a grid space of 0.375 Å^3^ centered on the alpha carbons of Tyr293 or Trp102, as mentioned previously. The 2D maps showing the receptor’s residues interacting with the ligands were obtained using the Discovery Studio Visualizer, version 24.1.0.23298 (https://discover.3ds.com/discovery-studio-visualizer-download; accessed 3 August 2024).

### 4.5. Structural Model of the LPA_3_ Receptor Inserted in the Membrane and Binding Site Detection

The structural model of the LPA_3_ receptor inserted in the membrane was generated using the AlphaFold3 prediction system [36]. Using the default modeling settings, the AlphaFold3 server (https://golgi.sandbox.google.com/; accessed 28 March 2025) was used to predict the receptor’s structure in a membrane-mimicking environment, including 100 lipid molecules. The identity and arrangement of the lipids were inferred from the model output, and no manual membrane assembly or insertion was required. The top-ranked model (based on internal confidence metrics such as pTM and ipTM scores) was selected for further analysis.

Ligand-binding sites were predicted using DeepSite [37] (accessed 28 March 2025), a protein-binding pocket predictor based on deep neural networks. The input protein structure (LPA_3_ model) was preprocessed to compute 3D voxel representations encoding physicochemical interaction channels. These features were evaluated using a pre-trained Deep Convolutional Neural Network (DCNN) model, executed with Graphics Processing Unit acceleration. DeepSite produced spatial probability maps highlighting regions on the protein surface with a high likelihood of ligand binding (cutoff score: >0.95). These predictions guided subsequent analysis and visualization of potential binding pockets.

### 4.6. Reagents, Plasmids, and Cells

1-Oleyl lysophosphatidic acid (LPA) (https://pubchem.ncbi.nlm.nih.gov/compound/5311263#section=ChEBI-ID; accessed 25 March 2025) and 2S-(1-oleoyl-2-O-methyl-glycerophosphothionate) (OMPT) (https://pubchem.ncbi.nlm.nih.gov/compound/5311263#section=ChEBI-ID; accessed 25 March 2025) were from Cayman Chemical Co. (Ann Arbor, MI, USA). Dulbecco’s modified Eagle’s medium, trypsin, Lipofectamine 2000, streptomycin, penicillin, amphotericin B, blasticidin, hygromycin B, doxycycline hyclate, and Fura-2 AM were purchased from Invitrogen-Life Technologies (Carlsbad, CA, USA). Our previous publications indicate the source of other materials [30,32,38].

Parental Flp-In T-Rex HEK293 cells were obtained from Invitrogen (Carlsbad, CA, USA). The LPA_3_ receptor sequence was fused at the carboxyl terminus (Ctail) with a green fluorescent protein and cloned into the pCDNA5/FRT/TO plasmid to employ the inducible Flp-In TREx expression system [30,32,38]. Mutants of this plasmid were obtained commercially (Bioinnovatise, Inc., Rockville, MD, USA), and the changes were confirmed by sequencing. Those amino acids identified to interact with the ligands in the docking simulations were substituted by alanine. Two main agonist-binding cavities were detected (Cavity 1 and 2, see Results). The residues replaced by alanines for the Upper Cavity mutant were (Thr25, Leu86, Thr90, Arg105, Gln106, Asp110, Leu113, Leu179, Trp191, Trp252, Gly255, Leu259, Lys275, Arg276, Phe278, and Leu279). For the Lower Cavity mutant, the amino acids substituted were (Ile131, Met134, Arg135, Val136, Val213, Lys216, Thr217, Val219, Leu220, Arg230, Pro234, Lys275, Arg276, and Phe278). Only the Lower Cavity’s last three amino acids (i.e., Lys275, Arg276, and Phe278) were present in the Upper Cavity. A third mutant was also obtained in which all the residues detected in these binding pockets were substituted by alanine (Upper + Lower Cavity mutant). T-Rex HEK293 cells were transfected with plasmids for the expression of the wild-type and mutant receptors and were subjected to selection as described before [30,32,38].

### 4.7. Intracellular Calcium Concentration

Determinations were performed as previously described [30,32,38]. In brief, the cells were serum-starved and treated for 12 h with 100 ng/mL doxycycline hyclate to induce LPA_3_ expression in all receptors (wild-type and mutants were tagged with the green fluorescent protein [30,32,38]; the density was similar as reflected by fluorescence microscopy). The cells were loaded with 2.5 μM Fura-2 AM for 1 h. Cells were carefully detached from the Petri dishes, washed to eliminate unincorporated dye, and maintained in suspension. Two excitation wavelengths (340 and 380 nm) and an emission wavelength of 510 nm were employed. Intracellular calcium levels were calculated as described by Grynkiewicz et al. [39].

### 4.8. Statistical Analyses

The data are presented as the means ± standard error of the means. Statistical analyses were performed using ordinary one-way ANOVA with the Bonferroni post-test using the software included in the GraphPad Prism program (version 10.2.3). A *p*-value < 0.05 was considered statistically significant.

## Figures and Tables

**Table 1:** Thermodynamic parameters and interacting amino acids were determined in the distinct docking simulations using LPA as the ligand.

Focus (Charge)	∆G (Kcal/mol)	Interacting Amino Acids
NONE (0)	−5.01	Val219, Leu220, Ser221, Lys216, Arg230, Pro234, Val136
NONE (−1)	−4.10	Arg276, Trp277, Val22, His273, Val274, Lys 29, Trp25, Ile 32, Leu 280, Val33, Val 36, Tyr83, Gly 37, Leu283, Met 87, Leu86, Cys41
NONE (−2)	−7.18	Lys216, Thr217, Ile131, Met134, Arg230, Pro234, Leu237, Arg127, Val136
Trp102 (0)	−5.99	Leu283, Leu280, Leu279, Met87, Val33, Thr90, Trp25, Arg276, Lys29
Trp102 (−1)	−4.10	Gln106, Arg105, Lys275, Leu86, Tyr14, Leu109, Trp102, Ala180, Leu279, Thr90, Asp23, Gly91, Arg276, Val36, Val33, Leu280, Trp277, Trp225, Lys29, Ile32
Trp102 (−2)	−8.93	Trp277, Leu279, Arg276, Gly37, Val33, Trp25, Lys29, Thr90, Leu86, Met87
Tyr293 (0)	−5.79	Leu220, Lys216, Thr217, Ile131, Val213, Pro234, Arg135, Met134, Val136, Asn139, His137, Phe63
Tyr293 (−1)	−2.88	Thr146, Lys142, Val145, Trp153, Ile149, Leu150, Tyr67, Val55, Ile56, His62, Ala70, Asn71
Tyr293 (−2)	−7.62	Trp277, Leu280, Leu279, Leu283, Tyr83, Arg276, Gly37, Val33, Trp25, Leu86, Met87

**Table 2:** Thermodynamic parameters and interacting amino acids were determined in the distinct docking simulations using OMPT as the ligand.

Focus (Charge)	∆G (Kcal/mol)	Interacting Amino Acids
ONE (0)	−5.67	Trp277, Leu279, Leu280, Leu283, Arg276, Tyr14, Asp23, Trp25, Ser94, Thr90, Arg105, Leu86, Val33
NONE (−1)	−5.36	Lys216, Val213, Ile131, Met134, Arg230, Val136, Arg135
NONE (−2)	−5.85	Ser94, Arg105, Leu86, Asp23, Lys275, Arg276, Leu279, Val36, Trp277, Val33, Ile32
Trp102 (0)	−5.15	Leu283, Leu280, Leu279, Tyr83. Val36, Met87, Val33, Thr90, Trp25, Arg276, Trp277, Leu86
Trp102 (−1)	−7.75	Leu256, Leu159, Leu188, Phe278, Lys275, Trp191, Leu179, Ala180, Tyr183, Tyr187, Gln106, Arg105, asp110, Ala282, Leu279, Trp252
Trp 102 (−2)	−7.11	Leu280, Leu279, Leu283, Ile32, Gly37, Val36, Val33, Arg276, Trp25, Met87
Tyr293 (0)	−4.64	Arg230, Leu220, Lys216, Thr217, Arg231, Ile131, His137, Val136, Thr233, Pro234
Tyr293 (−1)	−5.76	Arg232, Met235, Lys239, Lys236, Thr240, Tyr295, Glu298, Ser294, Tyr301, Ile291
Tyr293 (−2)	−5.64	Lys296, Lys236, Thr240, Lys305, Ile308, Tyr295, Glu298, Tyr301, Ser294, Met304,Ile291

## Data Availability

The data are available from the corresponding author upon reasonable request.

## Supplementary Materials

The following supporting information can be downloaded at https://www.mdpi.com/article/10.3390/ijms26094123/s1.
