# Supplementary material for: Multiple LPA3 Receptor Agonist Binding Sites Evidenced Under Docking and Functional Studies
Source: Int J Mol Sci. 2025 Apr 26;26(9):4123. doi: 10.3390/ijms26094123 (PMC12071260; doi:10.3390/ijms26094123)
Supplement: Supplementary file 1 [file ijms-26-04123-s001.zip › ijms-3543429-supplementary.pdf]

SUPPLEMENTARY MATERIAL

Supplementary Figure S1. Alignment of the FASTA sequence of the LPA_3 receptor (Q9UBY5) with the 3D model programs I-Tasser (model 01.pdb chainA_s001), Swiss Model (model1) and AlphaFold (AF-A v4).

5/11/24, 3:55 PM PROMALS3D Result

Colored PROMALS3D alignment (sequences in aligned order)

```
Conservation:                        99999999 999999999 99 9 9 99 999999 999999999 999999999 99 999 9999999
AF-A0A1W7RGA1-F1-model_v4      1     MNECHYDKRMDFFYNRSNADTADEWSGTTLVIVLCFGTFFCLFIFLSNSLVIAAVVKNKKFHYPFYYLLA   70
sp_Q9UBY5_LPAR3_HUMAN_Lys      1     MNECHYDKHMDFFYNRSNTDTVDDWTGTKLVIVLCVGTFFCLFIFFSNSLVIAAVIKNRKFHFPFYYLLA   70
model_01.pdb_chainA_s001       1     MNECHYDKHMDFFYNRSNTDTVDDWTGTKLVIVLCVGTFFCLFIFFSNSLVIAAVIKNRKFHFPFYYLLA   70
model1.pdb_chainA_s002         1     MNECHYDKHMDFFYNRSNTDTVDDWTGTKLVIVLCVGTFFCLFIFFSNSLVIAAVIKNRKFHFPFYYLLA   70
Consensus_aa:                        MNECHYDK+MDFFYNRSNhDThD-WoGTpLVIVLChGTFFCLFIFhSNSLVIAAVlKN+KFH@PFYYLLA
Consensus_ss:                                 eeee                hhhhhhhhhhhhhhhhhhhhhhhhhhh       hhhhhhh

Conservation:                        999999999999999999999999 99 999999999999 999999 9999999999  99 9999999
AF-A0A1W7RGA1-F1-model_v4     71     NLAAADFFAGIAYVFLMFNTGPVSRTLSVNRWFLRQGLLDTSLTASLANLLVIAVERHISIVKMRVHSNL  140
sp_Q9UBY5_LPAR3_HUMAN_Lys     71     NLAAADFFAGIAYVFLMFNTGPVSKTLTVNRWFLRQGLLDSSLTASLTNLLVIAVERHMSIMRMRVHSNL  140
model_01.pdb_chainA_s001      71     NLAAADFFAGIAYVFLMFNTGPVSKTLTVNRWFLRQGLLDSSLTASLTNLLVIAVERHMSIMRMRVHSNL  140
model1.pdb_chainA_s002        71     NLAAADFFAGIAYVFLMFNTGPVSKTLTVNRWFLRQGLLDSSLTASLTNLLVIAVERHMSIMRMRVHSNL  140
Consensus_aa:                        NLAAADFFAGIAYVFLMFNTGPVS+TLoVNRWFLRQGLLDoSLTASLhNLLVIAVERHhSIh+MRVHSNL
Consensus_ss:                        hhhhhhhhhhhhhhhhhhhhhhhhhhhhhhhhhhhhhhhhhhhhhhhhhhhhhhhhhhhhhh

Conservation:                        999999 9999 9999999999999999999 99 9999999999999 9999999  9 999999 999
AF-A0A1W7RGA1-F1-model_v4    141     TKKRVTFLILLIWAIAIFMGAVPTLGWNCLCDISTCSSLAPIYSRSYLIFWTVSNLGVFFIMVVVYIRIY  210
sp_Q9UBY5_LPAR3_HUMAN_Lys    141     TKKRVTLLILLVWAIAIFMGAVPTLGWNCLCNISACSSLAPIYSRSYLVFWTVSNLMAFLIMVVVYLRIY  210
model_01.pdb_chainA_s001     141     TKKRVTLLILLVWAIAIFMGAVPTLGWNCLCNISACSSLAPIYSRSYLVFWTVSNLMAFLIMVVVYLRIY  210
model1.pdb_chainA_s002       141     TKKRVTLLILLVWAIAIFMGAVPTLGWNCLCNISACSSLAPIYSRSYLVFWTVSNLMAFLIMVVVYLRIY  210
Consensus_aa:                        TKKRVThLILLlWAIAIFMGAVPTLGWNCLCsIShCSSLAPIYSRSYLlFWTVSNL.hFhIMVVVYlRIY
Consensus_ss:                            hhhhhhhhhhhhhhhhhhhh             eeeeee   hhheeehhhhhhhhhhhhhhhhhh

Conservation:                         99 99999999999999999 9999999999 99999 99999999999999999  9 99 9999999
AF-A0A1W7RGA1-F1-model_v4    211     MYVQRKTNVLSPHTSGSISRRKTPMKLMKTVMAVLGAFIVCWTPGLVVLLLDGLNCTRCEVQNVKRWFLL  280
sp_Q9UBY5_LPAR3_HUMAN_Lys    211     VYVKRKTNVLSPHTSGSISRRRTPMKLMKTVMTVLGAFVVCWTPGLVVLLLDGLNCRQCGVQHVKRWFLL  280
model_01.pdb_chainA_s001     211     VYVKRKTNVLSPHTSGSISRRRTPMKLMKTVMTVLGAFVVCWTPGLVVLLLDGLNCRQCGVQHVKRWFLL  280
model1.pdb_chainA_s002       211     VYVKRKTNVLSPHTSGSISRRRTPMKLMKTVMTVLGAFVVCWTPGLVVLLLDGLNCRQCGVQHVKRWFLL  280
Consensus_aa:                        hYVpRKTNVLSPHTSGSISRR+TPMKLMKTVMhVLGAFlVCWTPGLVVLLLDGLNCppC.VQpVKRWFLL
Consensus_ss:                        hhhhhhhhhhhh     hhhhhhhhhhhhhhhhhhhhhhhhhhhhhhhhhhh         hhhhhhhhh

Conservation:                        999999  999999 99  9  99   9999 9       99   9999 99 99 9  999 9  999
AF-A0A1W7RGA1-F1-model_v4    281     LALLNSLMNPIIYSCKDDEMSNTMRRMICCSFEERSQERWSLRIPSAVLGRSIDSSSQYLENGISQVTIS  350
sp_Q9UBY5_LPAR3_HUMAN_Lys    281     LALLNSVVNPIIYSYKDEDMYGTMKKMICC-FSQENPERRPSRIPSTVLSRS-DTGSQYIEDSISQGAVC  348
model_01.pdb_chainA_s001     281     LALLNSVVNPIIYSYKDEDMYGTMKKMICC-FSQENPERRPSRIPSTVLSRS-DTGSQYIEDSISQGAVC  348
model1.pdb_chainA_s002       281     LALLNSVVNPIIYSYKDEDMYGTMKKMICC-FSQENPERRPSRIPSTVLSRS-DTGSQYIEDSISQGAVC  348
Consensus_aa:                        LALLNSlhNPIIYShKD--M.sTM++MICC.Fppcs.ER.s.RIPShVLtRS.DotSQYlEstISQshlt
Consensus_ss:                        hhhhhhhhhhhhhhh  hhhhhhhhhhhhh                                 eeeeee

Conservation:                         9
AF-A0A1W7RGA1-F1-model_v4    351     GKENT  355
sp_Q9UBY5_LPAR3_HUMAN_Lys    349     NKSTS  353
model_01.pdb_chainA_s001     349     NKSTS  353
model1.pdb_chainA_s002       349     NKSTS  353
Consensus_aa:                        sKpso
Consensus_ss:
```

Supplementary Figure S2. Images resulting from the LPA_3-LPA (blue) and LPA_3-OMPT (red) docking analysis using PyMol.

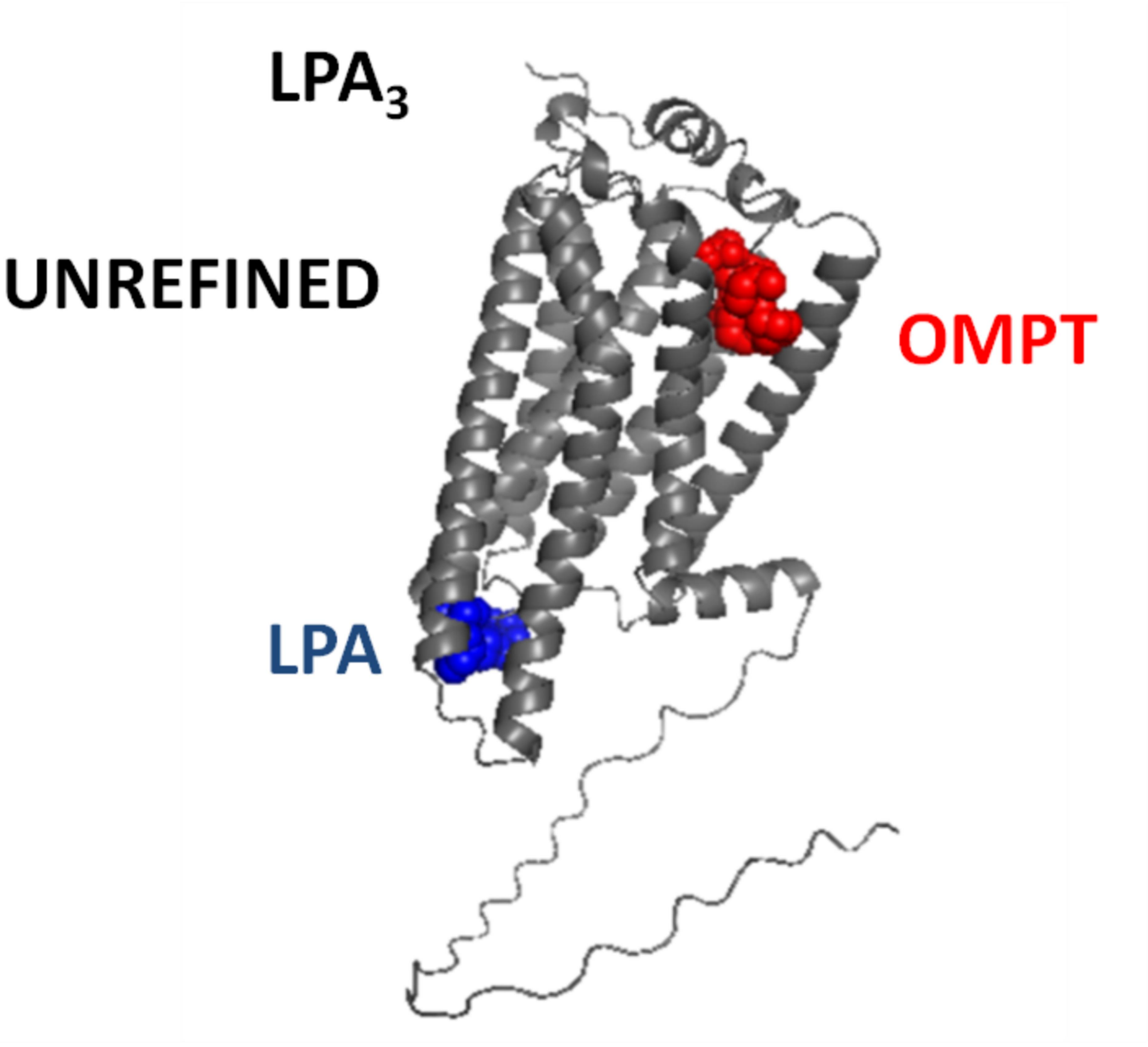

Supplementary Figure S3. Cartoon showing the LPA_3 receptor sites interacting with LPA (blue) and OMPT (red).

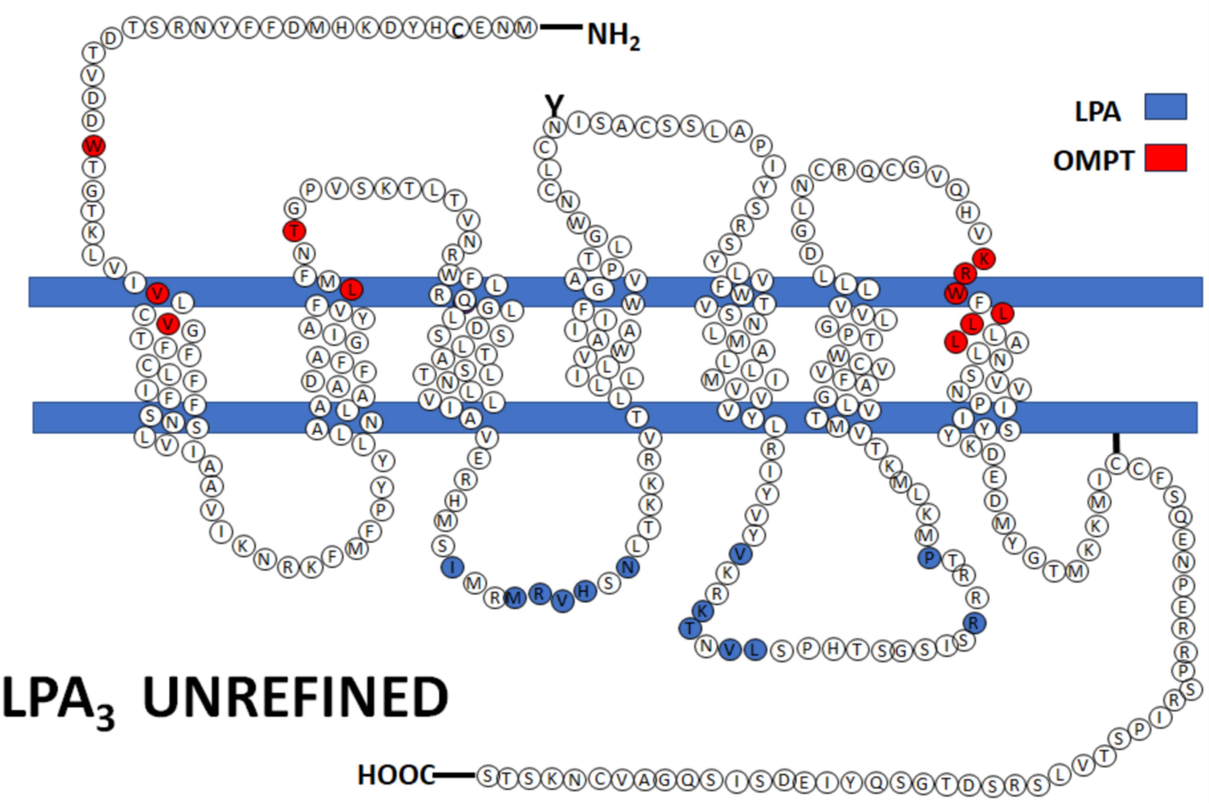

Supplementary Figure S4. The 2D diagram shows interactions observed at the LPA_3 binding site (Lower Cavity) with LPA (Discovery Studio Visualizer); critical amino acids contributing to interactions are shown in circles.

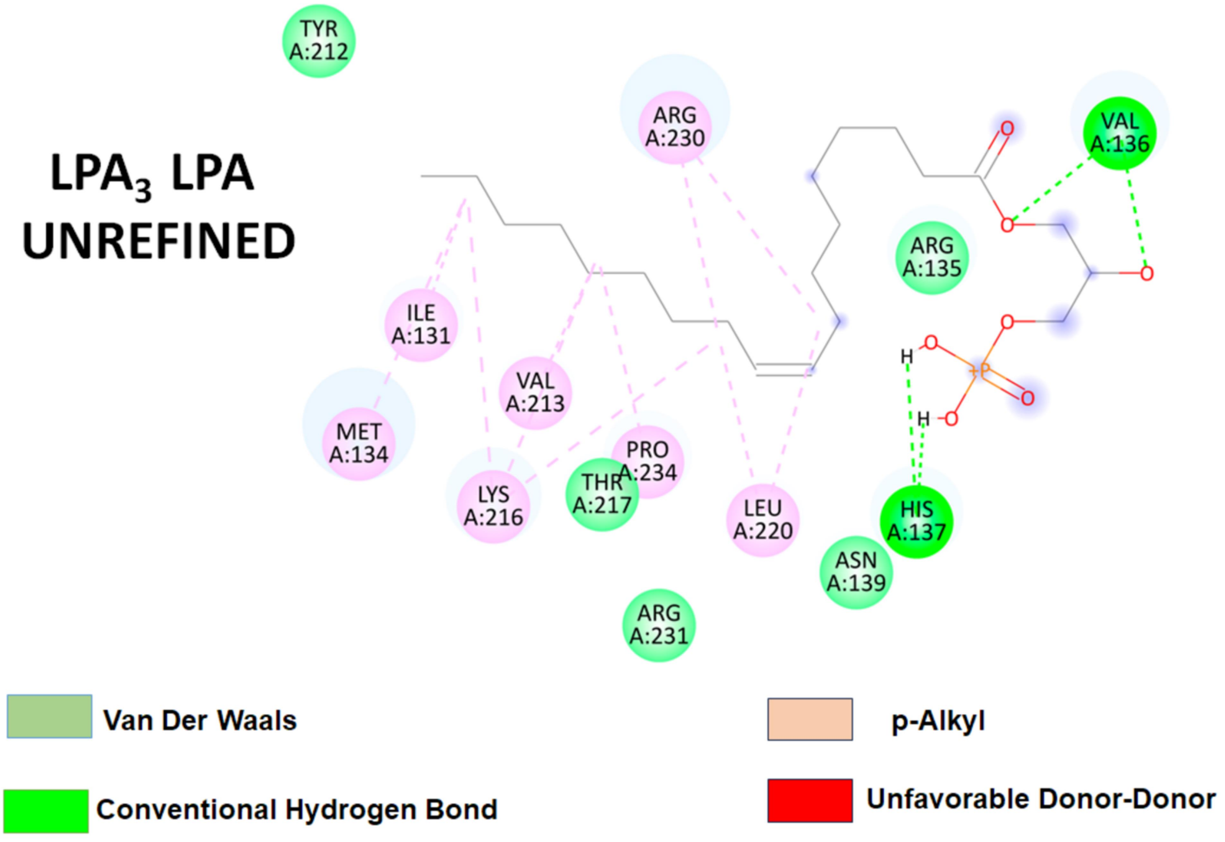

Supplementary Figure S5. The 2D diagram shows interactions at the LPA_3 binding site (Upper Cavity) with OMPT (Discovery Studio Visualizer). Critical amino acids contributing to interactions are shown in circles.

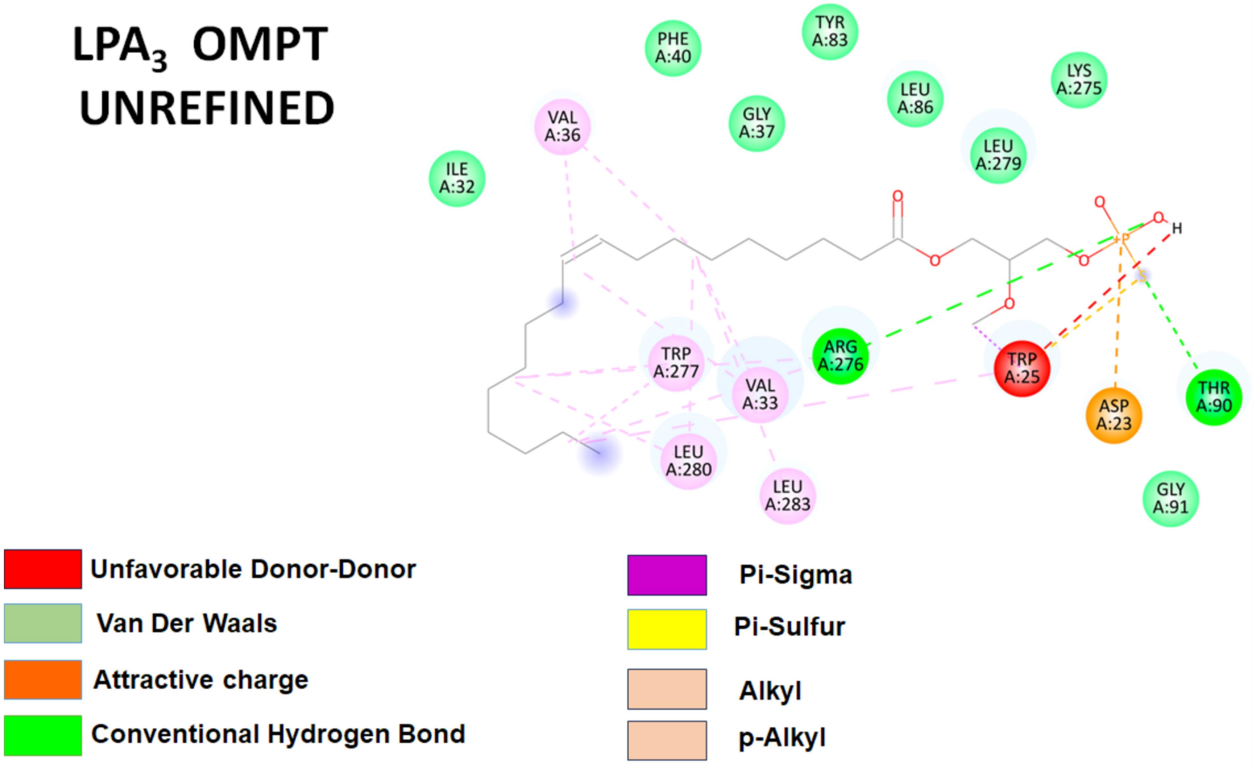

Supplementary Figure S6. Cartoons showing the refined LPA_3 receptor sites interacting with LPA (blue) and OMPT (red). Charges are indicated in the Figures.

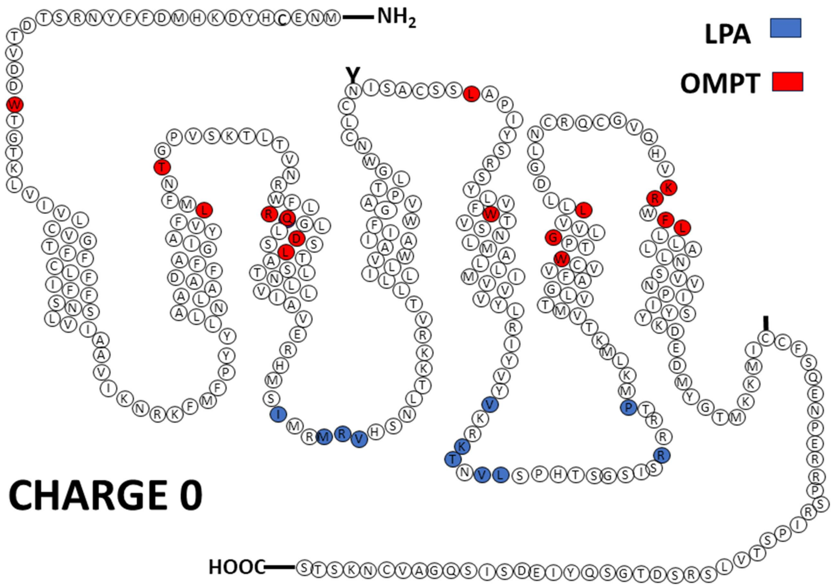

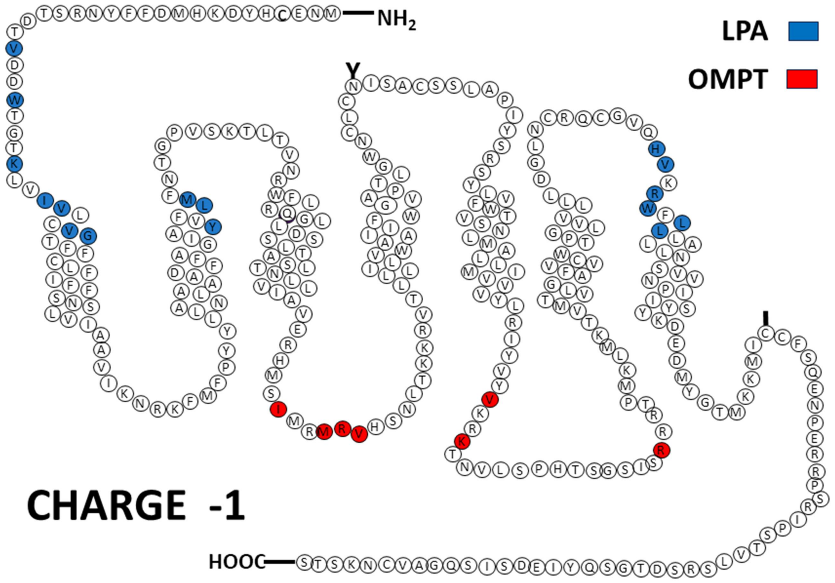
NH2
LPA
OMPT
CHARGE -1
HOOC

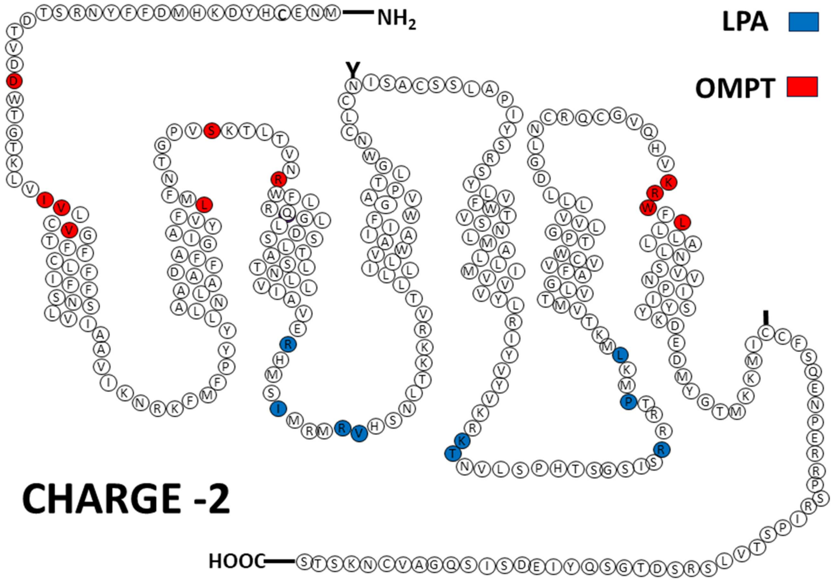
NH2
LPA
OMPT
CHARGE -2
HOOC

Supplementary Figure S7. The 2D diagram shows interactions at the LPA_3 binding site with LPA or OMPT. In the individual images, Ligand and Charge are indicated (Discovery Studio Visualizer). Critical amino acids contributing to interactions are shown in circles.

LPA(0)

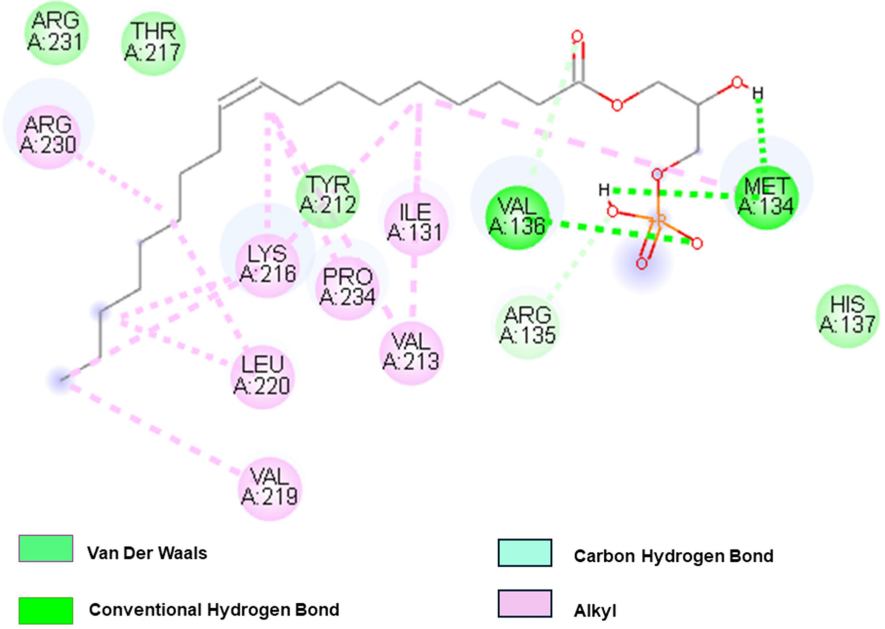

LPA(-1)

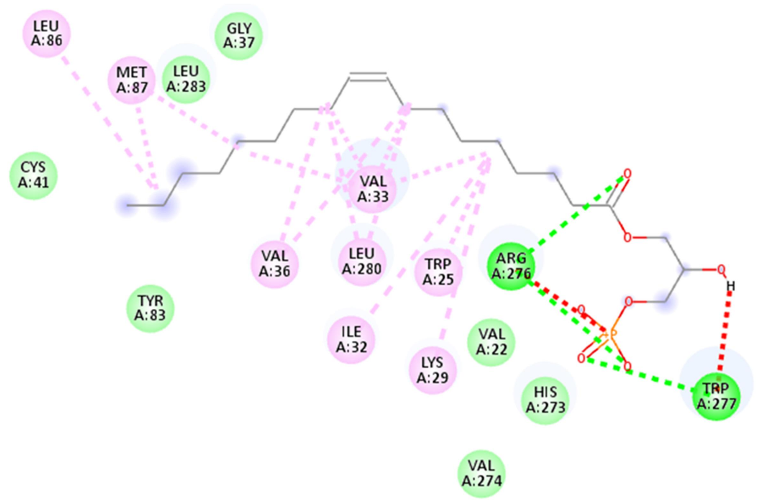

Unfavorable Positive-Positive

Van Der Waals

Unfavorable Donor-Donor

Conventional Hydrogen Bond

Carbon Hydrogen Bond

Alkyl

Pi- Alkyl

LPA(-2)

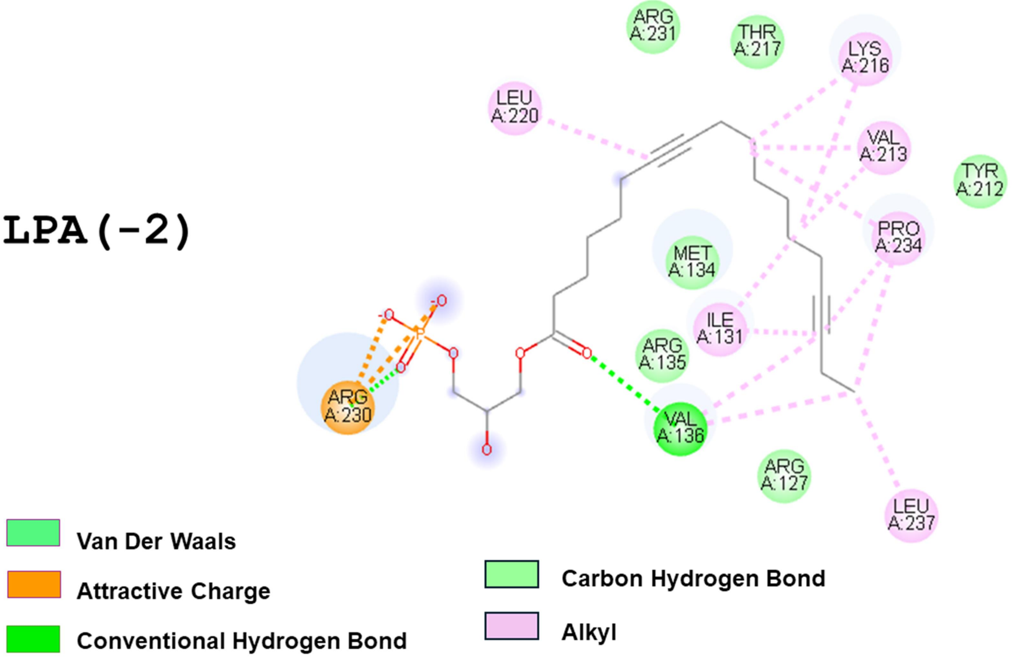
ARG A:231
THR A:217
LYS A:216
LEU A:220
VAL A:213
TYR A:212
PRO A:234
MET A:134
ILE A:131
ARG A:135
ARG A:230
VAL A:136
ARG A:127
LEU A:237
Van Der Waals
Attractive Charge
Conventional Hydrogen Bond
Carbon Hydrogen Bond
Alkyl

OMPT (0)

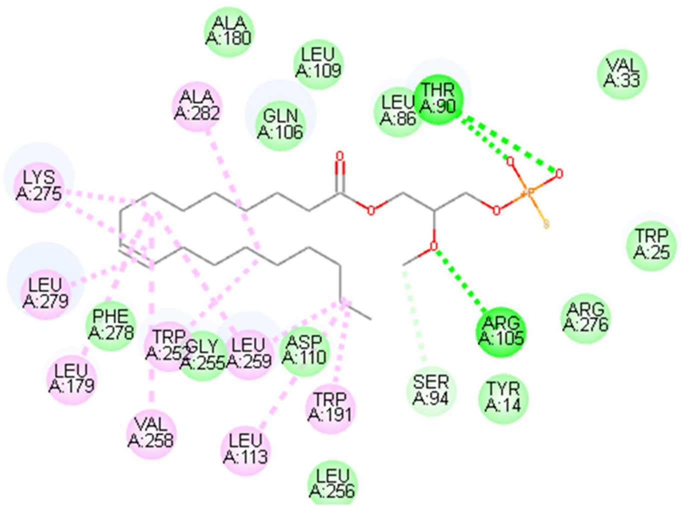

Van Der Waals

Conventional Hydrogen Bond

Carbon Hydrogen Bond

Alkyl

Pi-Alkyl

OMPT(-1)

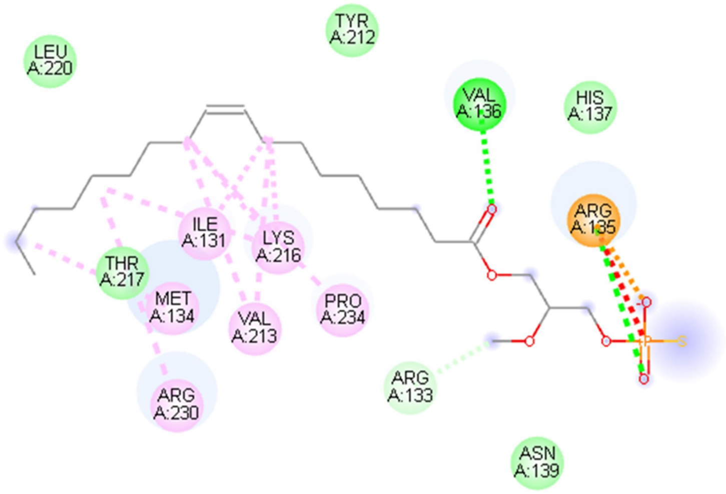
LEU A:220
TYR A:212
VAL A:136
HIS A:137
ARG A:135
ILE A:131
LYS A:216
THR A:217
MET A:134
VAL A:213
PRO A:234
ARG A:133
ARG A:230
ASN A:139

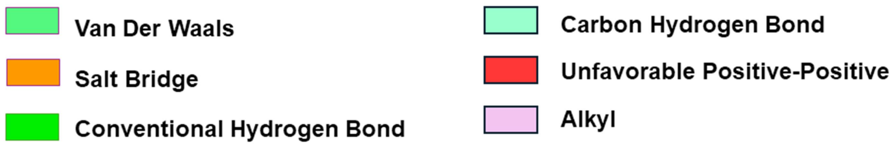
Van Der Waals
Salt Bridge
Conventional Hydrogen Bond
Carbon Hydrogen Bond
Unfavorable Positive-Positive
Alkyl

OMPT (-2)

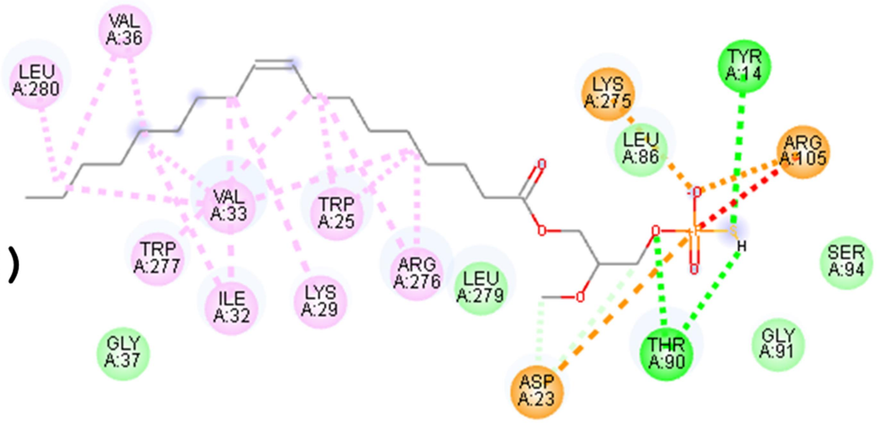

Van Der Waals

Salt Bridge

Attractive Charge

Conventional Hydrogen Bond

Carbon Hydrogen Bond

Unfavorable Positive-Positive

Alkyl

Pi-Alkyl

Supplementary Figure S8. Localization of the amino acids at the LPA_3 receptor to be focused. Trp102, orange; Tyr293, green.

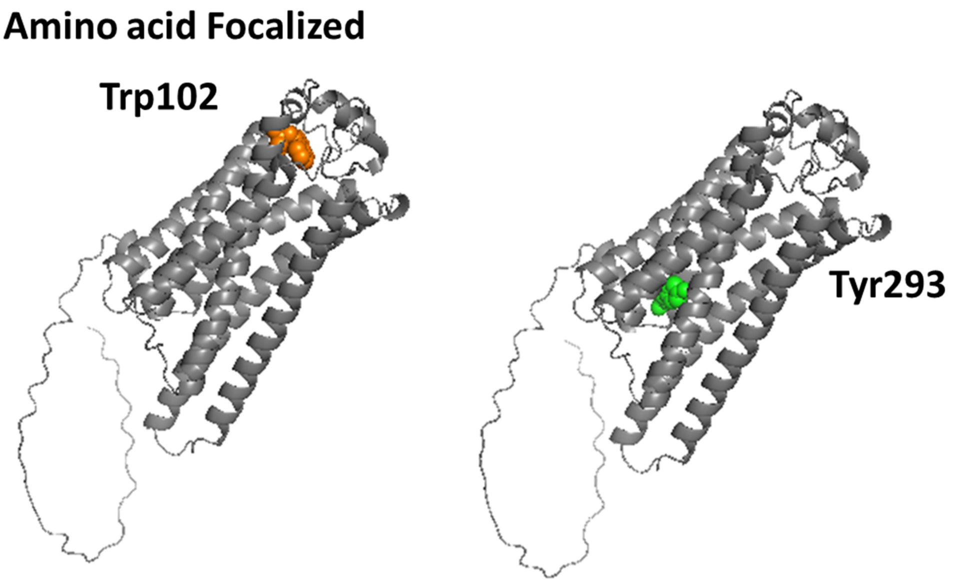

Supplementary Figure S9. Cartoons showing the LPA_3 receptor (Trp102-focused) sites interacting with LPA (blue) and OMPT (red). Charges are indicated in the Figures.

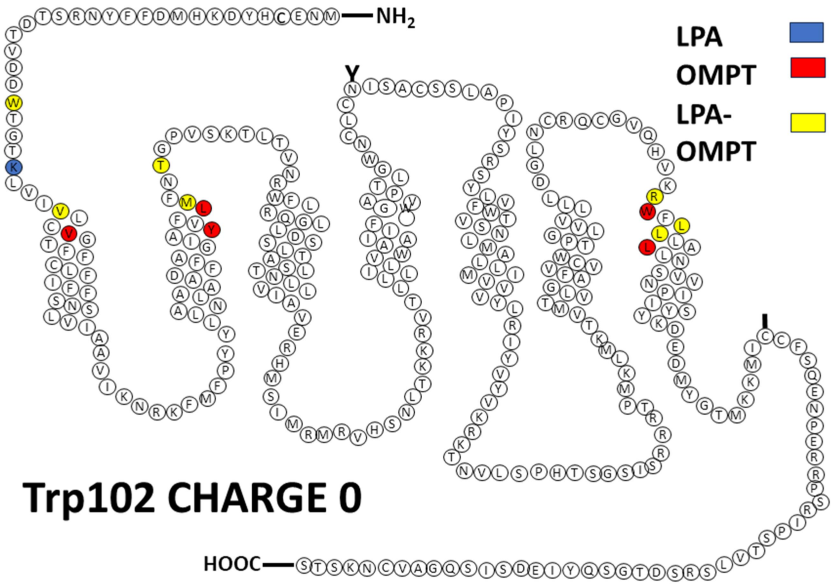

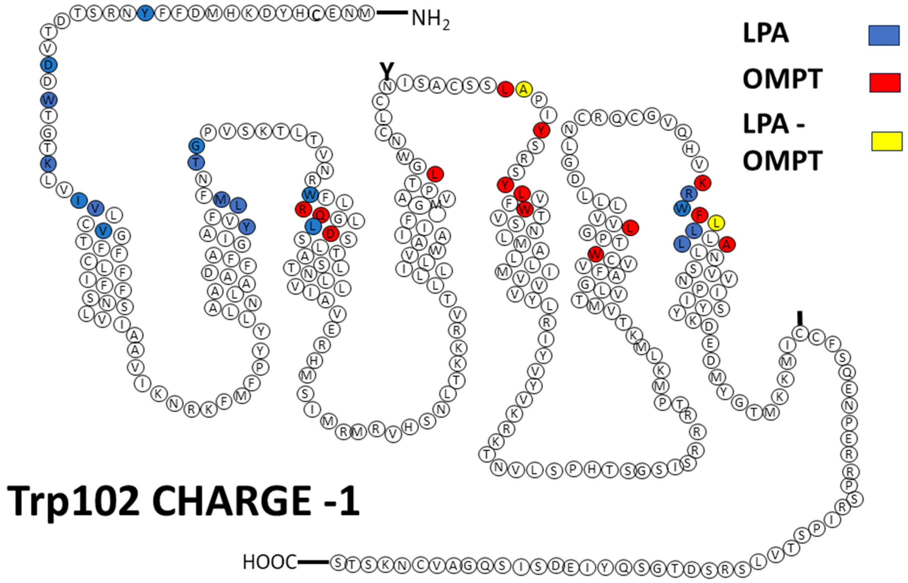
LPA
OMPT
LPA - OMPT
Trp102 CHARGE -1
NH2
HOOC

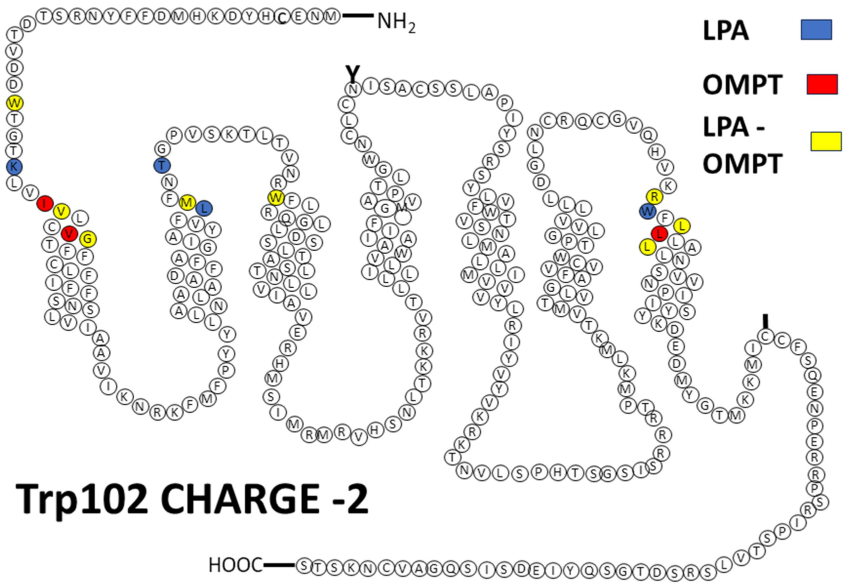
NH2
HOOC
LPA
OMPT
LPA - OMPT
Trp102 CHARGE -2

Supplementary Figure S10. The 2D diagram shows interactions of the LPA_3 (Trp102) structure with LPA or OMPT. In the individual images, Ligand and Charge are indicated (Discovery Studio Visualizer). Critical amino acids contributing to interactions are shown in circles.

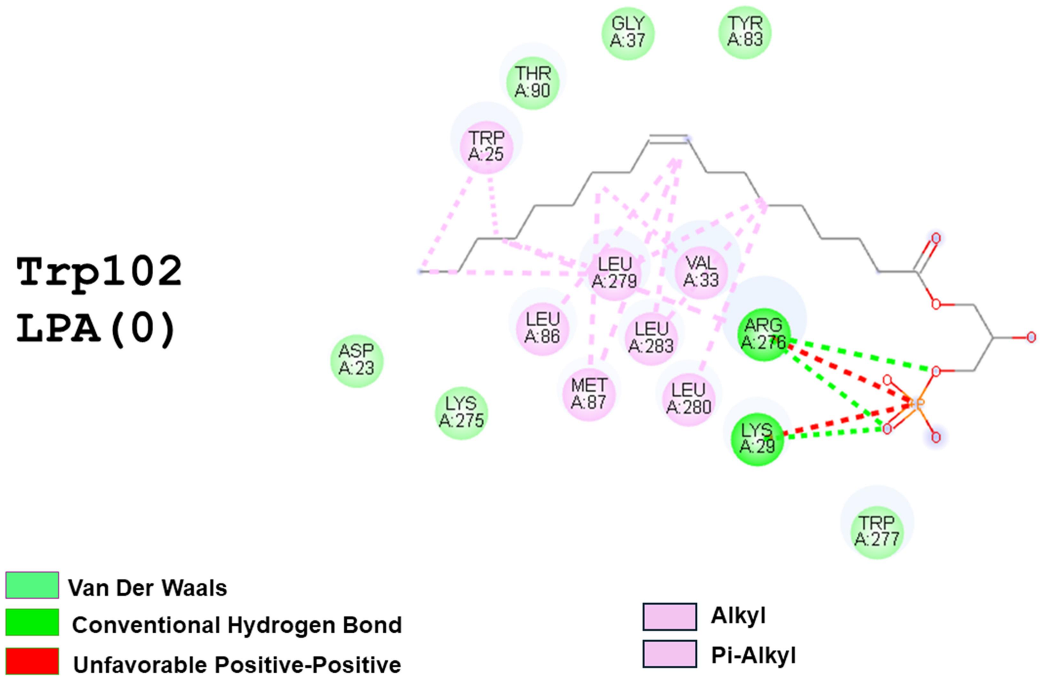

Trp102
LPA(-1)

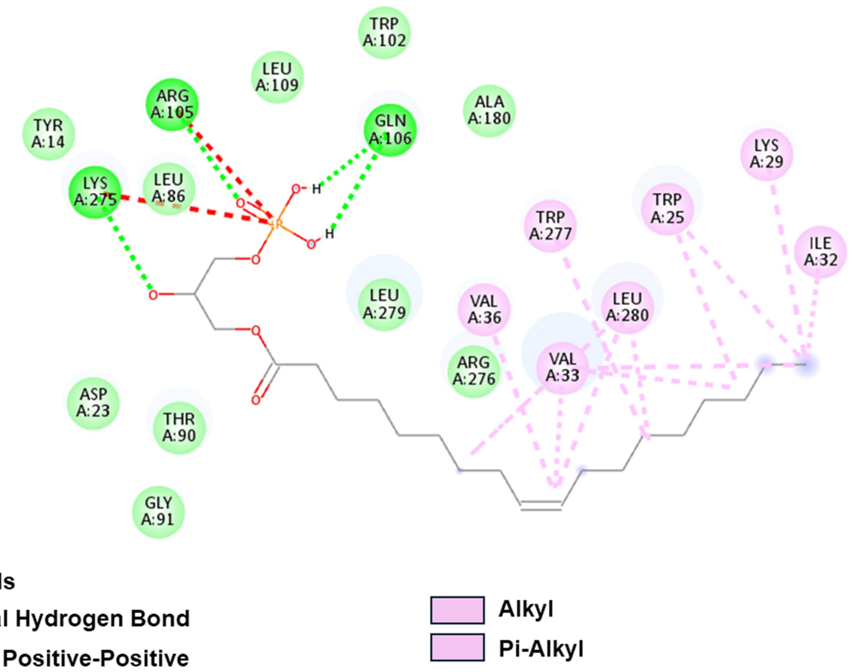
TRP A:102
LEU A:109
ARG A:105
GLN A:106
ALA A:180
TYR A:14
LYS A:275
LEU A:86
LYS A:29
TRP A:25
TRP A:277
ILE A:32
LEU A:279
VAL A:36
LEU A:280
VAL A:33
ARG A:276
ASP A:23
THR A:90
GLY A:91
Van Der Waals
Conventional Hydrogen Bond
Unfavorable Positive-Positive
Alkyl
Pi-Alkyl

Trp102
LPA(-2)

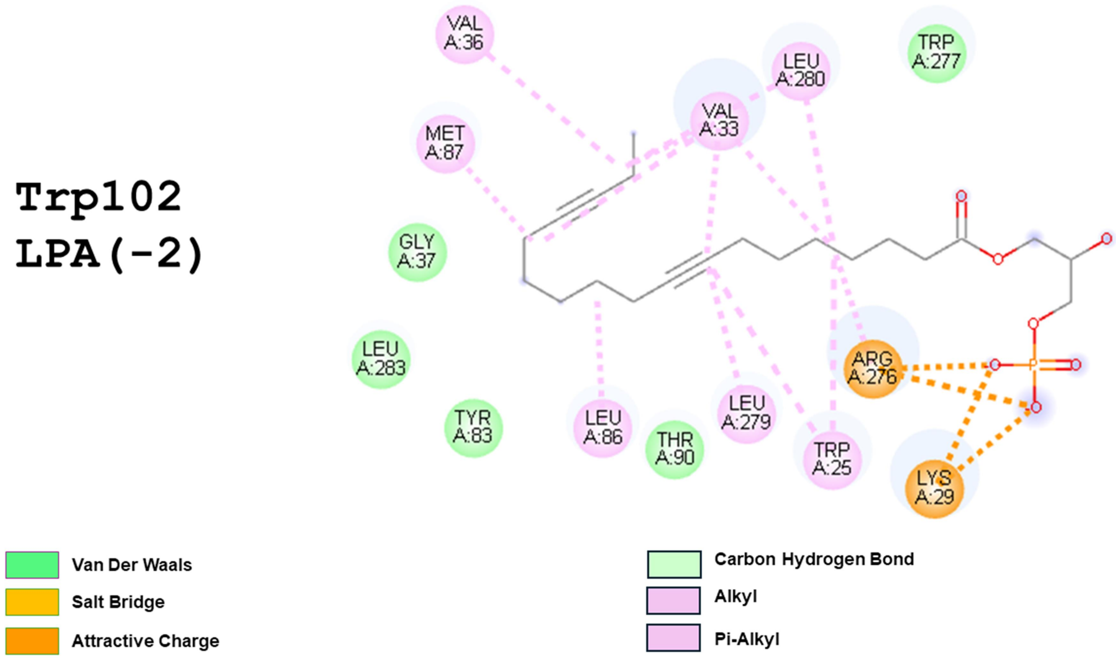
VAL A:36
LEU A:280
TRP A:277
VAL A:33
MET A:87
GLY A:37
LEU A:283
ARG A:276
TYR A:83
LEU A:86
THR A:90
LEU A:279
TRP A:25
LYS A:29
Van Der Waals
Salt Bridge
Attractive Charge
Carbon Hydrogen Bond
Alkyl
Pi-Alkyl

Trp102
OMPT(0)

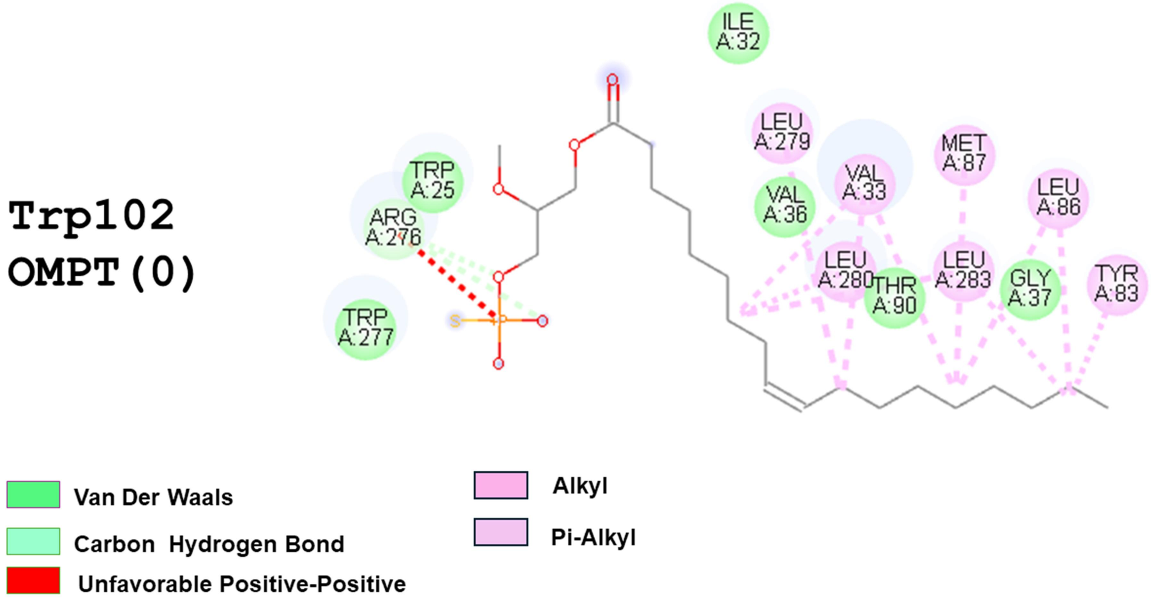

Trp102
OMPT(-1)

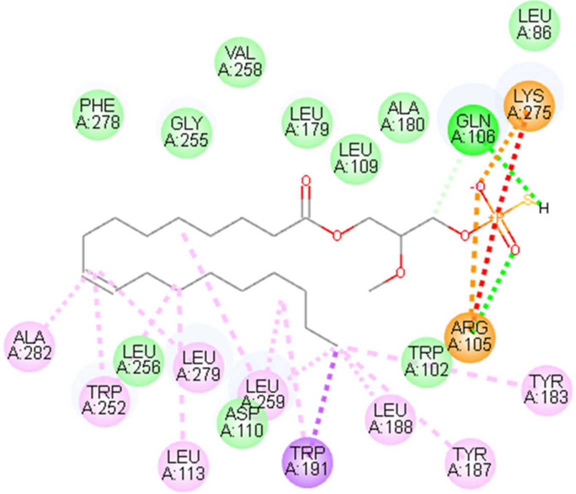

- Van Der Waals
- Attractive Charge
- Conventional Hydrogen Bond
- Carbon Hydrogen Bond
- Unfavorable Positive-Positive
- Pi-Sigma
- Alkyl
- Pi-Alkyl

Trp102
OMPT(-2)

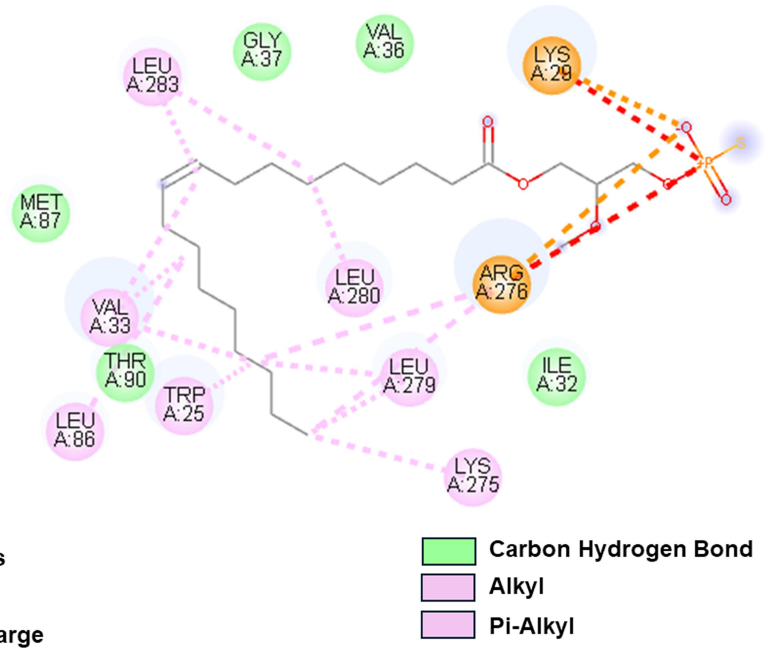

Supplementary Figure S11. Cartoons showing the LPA$_3$ receptor (Tyr293-focused) sites interacting with LPA (blue) and OMPT (red). Charges are indicated in the Figures.

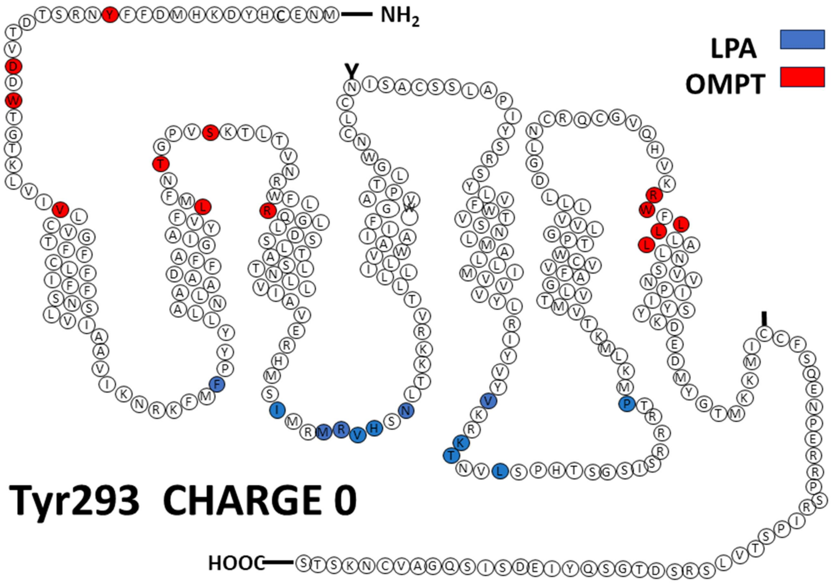

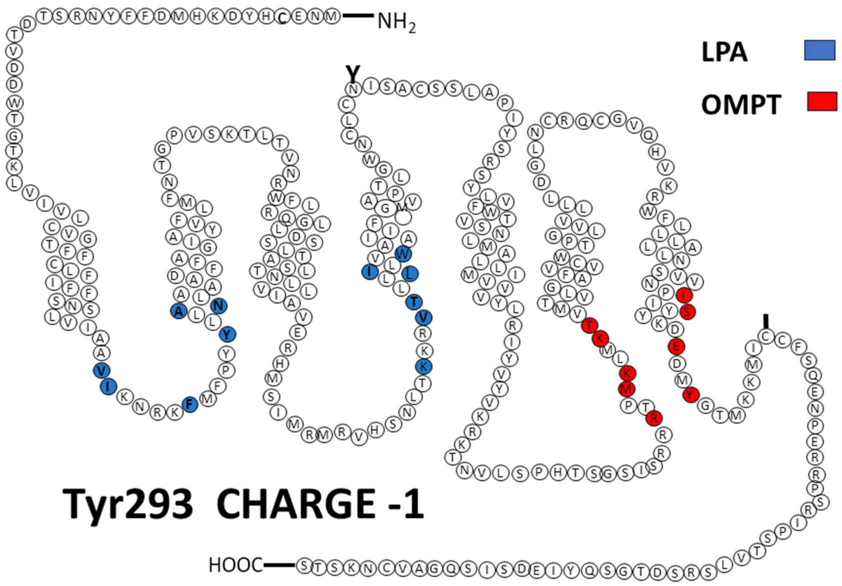
NH2
LPA
OMPT
Tyr293 CHARGE -1
HOOC

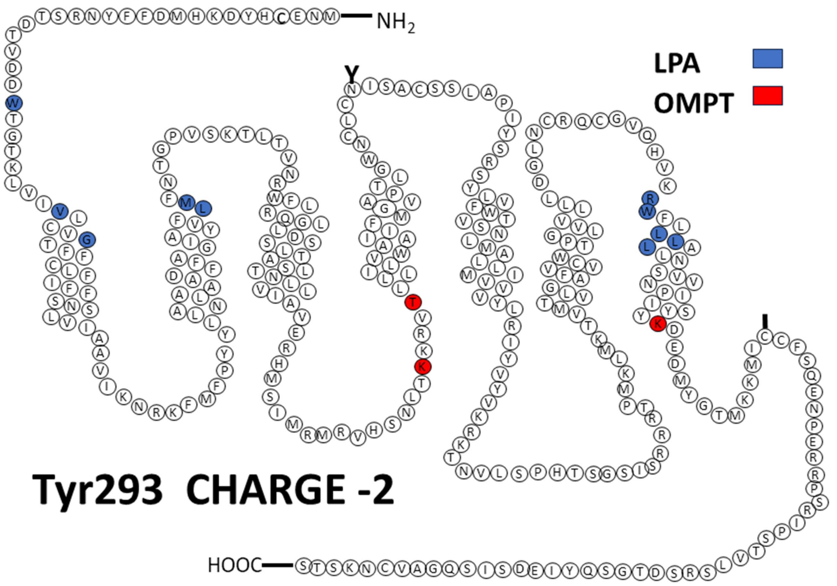
NH2
Y
LPA
OMPT
Tyr293 CHARGE -2
HOOC

Supplementary Figure S12. The 2D diagram shows interactions of the LPA_3 (Tyr293) structure with LPA or OMPT. In the individual images, Ligand and Charge are indicated (Discovery Studio Visualizer). Critical amino acids contributing to interactions are shown in circles.

Tyr293
LPA(0)

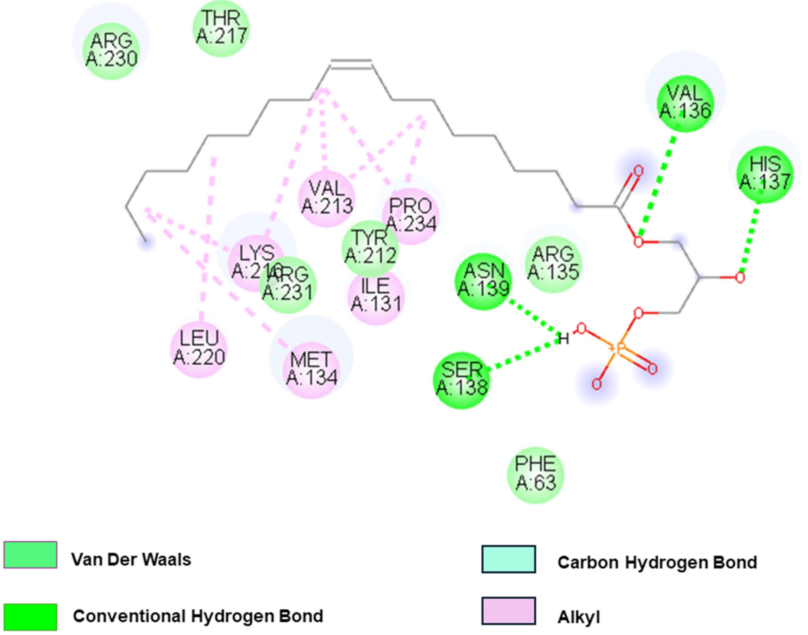

Tyr293
LPA(-1)

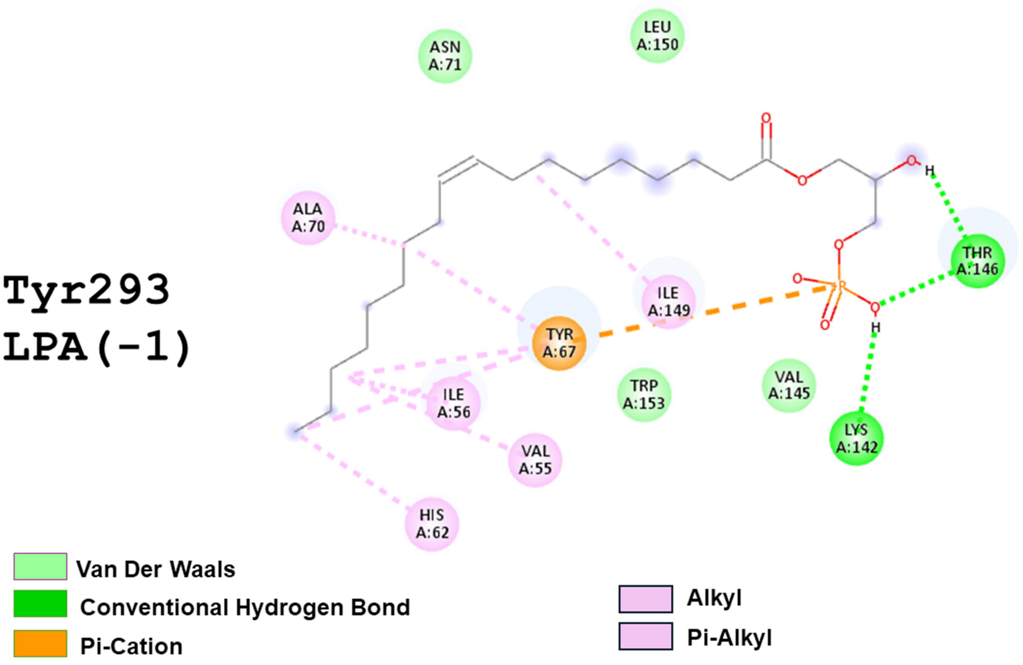
ASN A:71
LEU A:150
ALA A:70
ILE A:149
TYR A:67
THR A:146
ILE A:56
TRP A:153
VAL A:145
LYS A:142
VAL A:55
HIS A:62
Van Der Waals
Conventional Hydrogen Bond
Pi-Cation
Alkyl
Pi-Alkyl

Tyr293
LPA(-2)

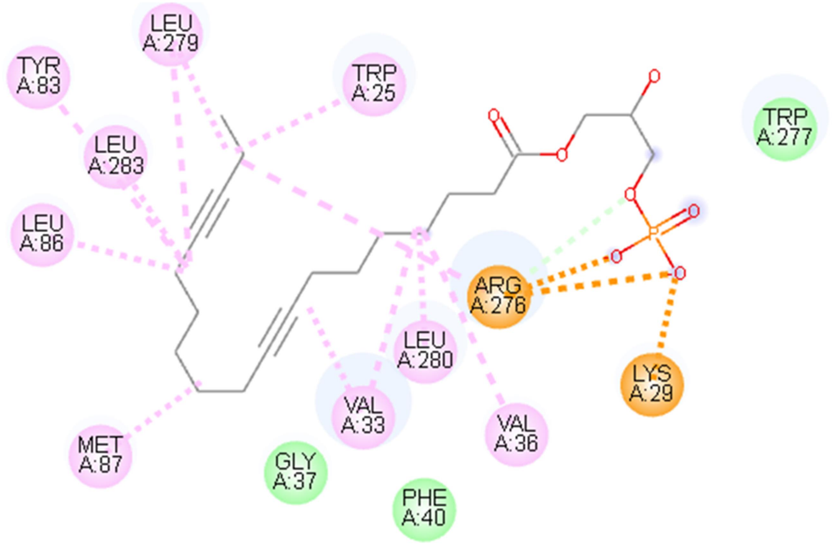

Van Der Waals
Salt Bridge
Attractive charge
Carbon Hydrogen Bond
Pi-Alkyl
Alkyl

Tyr 293
OMPT(0)

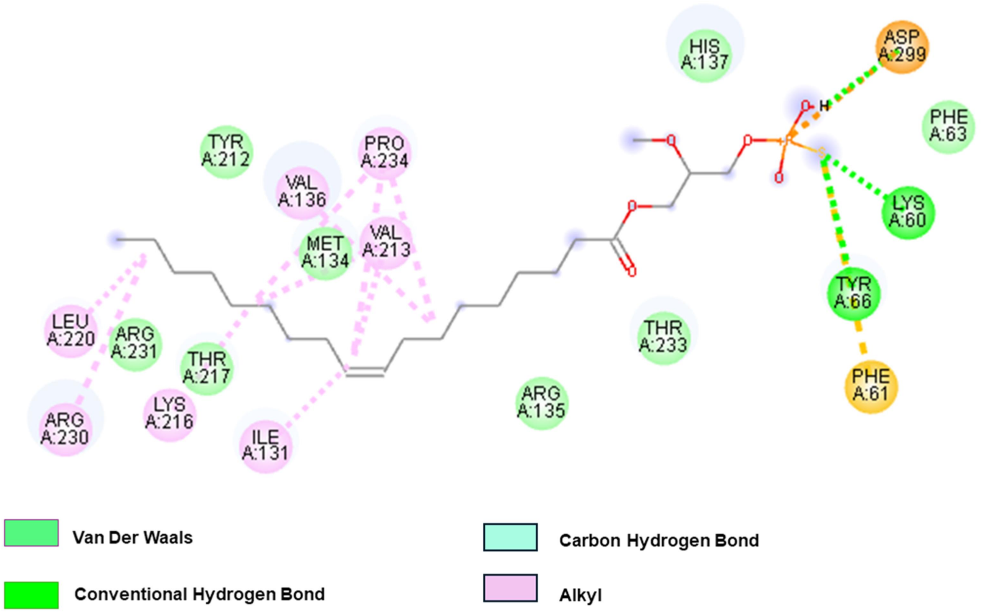

Tyr293
OMPT(-1)

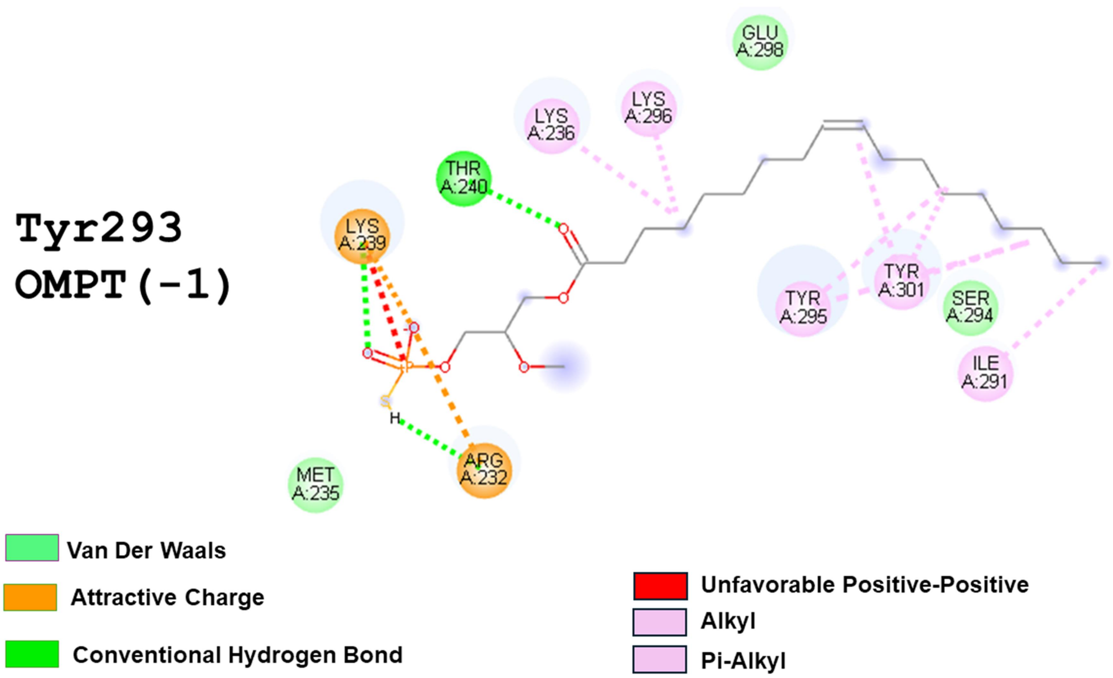

Tyr293
OMPT(-2)

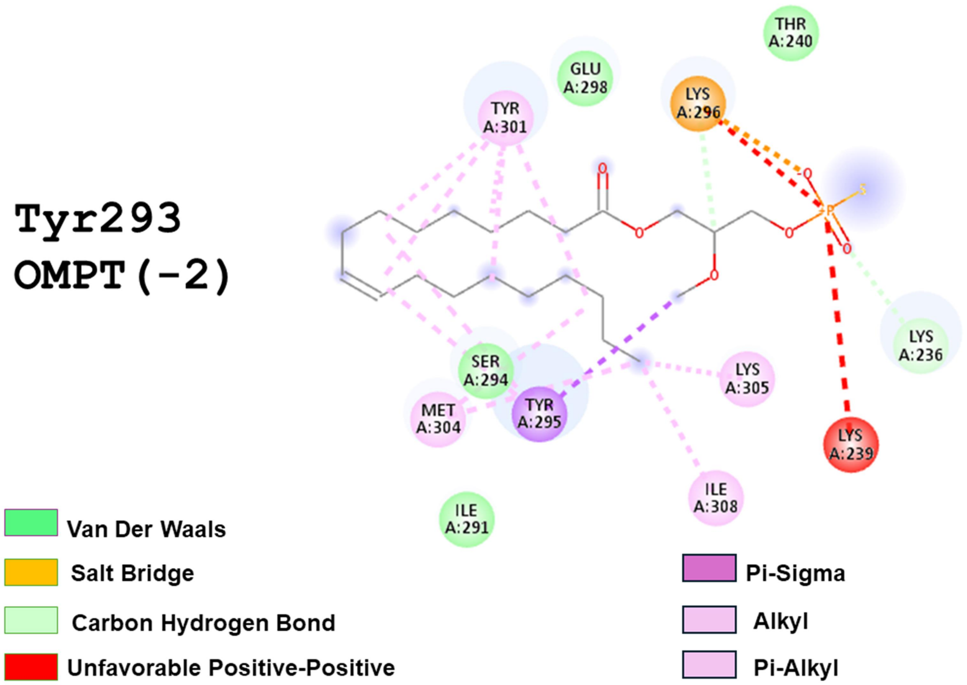

Supplementary Figure S13. Calcium tracings of cells transfected with the LPA_3 receptors that were or were not induced. In panel A, cells were stimulated with 1 μM LPA, whereas in panel B, 1 μM OMPT was the stimulus. Where indicated, 1 μM Ki16425 was added before the agonist. Data are representative of 3 experiments performed on different days with essentially identical results.

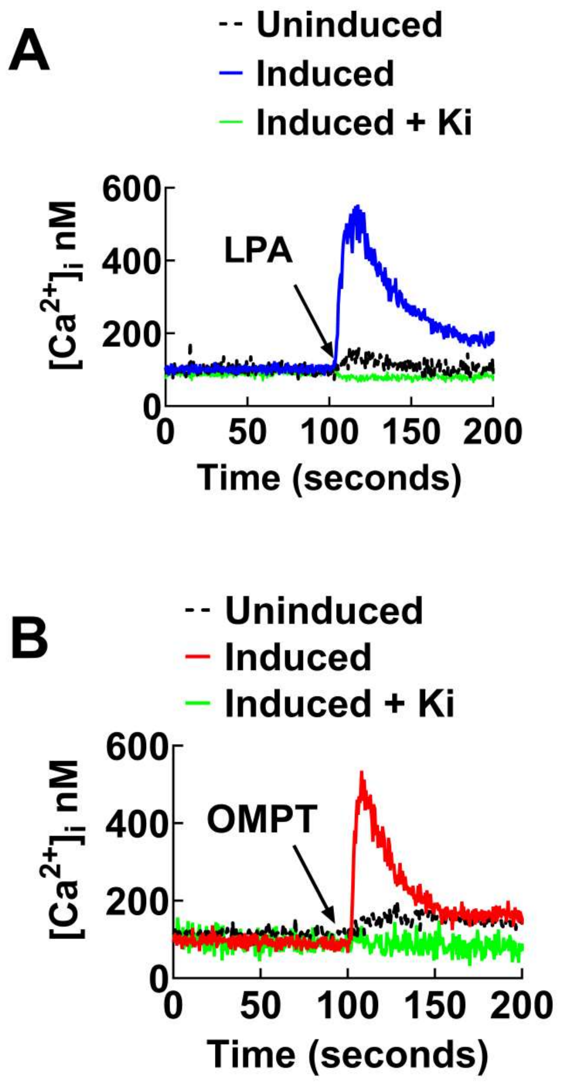

Supplementary Figure S14. LPA_3 receptor cavities were evidenced using PyMol. In the left panel, the cavities found are indicated in yellow. In the middle (Trp102-focused, Charge -1) and right (Trp102-focused, Charge -1) panel, the position of the ligands (LPA, blue, and OMPT, red) are indicated. The change in ligand color is due to merging with the cavity's yellow color.

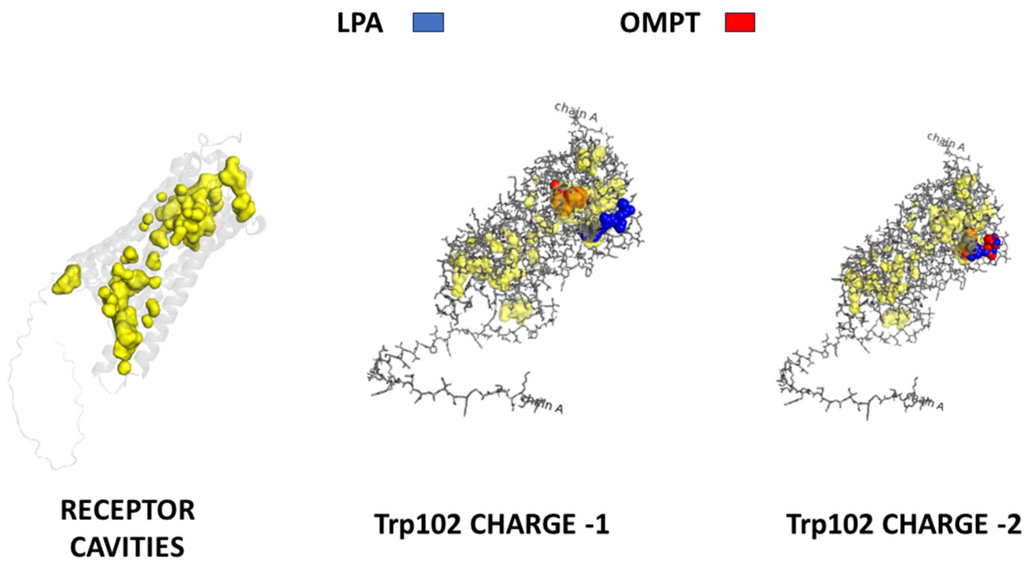

Supplementary Table S1. Structural evaluation of the 3D models generated using the three most frequently used homology modeling servers (in green, the best values).

Server	MolProbity: Protein Geometry/ Ramachandran favored	SAVESv6.0: ERRAT	SAVESv6.0: VERIFY
SWISS-MODEL	91.45%	94.8052	33.14%
I-TASSER	78.92%	83.4302	38.81%
AlphaFold	95.18%	94.3038	29.01%

Supplementary Table S2. Blind docking for LPA and OMPT at the unrefined LPA_3 receptor structure with a 126 Å3 grid box centered on the protein.

Ligand	ΔG (kcal/mol)	Sites of ligand-receptor interaction
LPA	-3.98	Leu220, Thr217, Arg230, Lys216, Val213, Met134, Ile131, Arg135, Pro234, Asn139, Val136, His137
OMPT	-5.31	Leu86, Leu283, Leu279, Leu280, Lys275, Val33, Val36, Thr90, Trp25, Arg276, Trp277
